# Lost in recessions: youth employment and earnings in Spain

**DOI:** 10.1007/s13209-021-00244-6

**Published:** 2021-11-26

**Authors:** Samuel Bentolila, Florentino Felgueroso, Marcel Jansen, Juan F. Jimeno

**Affiliations:** 1 CEMFI, Madrid, Spain; 2 Fedea, Madrid, Spain; 3 Universidad Autónoma de Madrid, Madrid, Spain; 4 Banco de España, Universidad de Alcalá, Madrid, Spain

**Keywords:** Youth, Employment, Earnings, Scarring effects, J13, J24, J31

## Abstract

Young workers in Spain face the unprecedented impact of the Great Recession and the COVID-19 crisis in short sequence. Moreover, they have also experienced a deterioration in their employment and earnings over the last three decades. In this paper, we document this evolution and adopt a longitudinal approach to show that employment and earnings losses suffered by young workers during recessions are not made up in the subsequent expansions. We also estimate the size of the scarring effects of entering the job market in a recession for college-educated workers during their first decade in the labor market. Our empirical estimates indicate that while there is some evidence of scarring effects, the driving force is a trend worsening of youth labor market outcomes.

**Supplementary Information:**

The online version contains supplementary material available at 10.1007/s13209-021-00244-6.

## Introduction

Spanish youth face huge challenges due to the succession of two deep economic crises in less than a decade. In the Great Recession (2008–2013), the youth unemployment rates reached record levels and young workers also belong to the most affected groups in the current economic crisis caused by the COVID-19 pandemic.

Moreover, the delicate position of Spanish youth is not new. The Spanish labor market is a hostile environment for young workers, and none of the recent reforms implemented has improved the functioning of the youth labor market. In the words of Juan J. Dolado, a great economist, a close friend of ours, and a movie buff, Spain is “no country for young people”. A couple of figures make this evident. Over the period 1983–2019, the average unemployment rate for workers aged 20–24 years old was equal to 32.7%, and for those aged 25–29 years old, it was equal to 22.3%. In contrast, in the (then) European Union of 28 countries (EU-28), the corresponding averages were, respectively, 17.8% and 11.5%, in other words, about half the size of the rates in Spain. Furthermore, these poor numbers are not simply the result of the overall unemployment rate being significantly higher in Spain than elsewhere in Europe. Young Spaniards suffer on every front: they have low employment rates, high rates of temporary employment with correspondingly high levels of job rotation and mismatch, and low wages.

The economic analysis of the causes of low employment rates and high unemployment rates among young workers can be divided into two strands of research. The first one studies the relevance of education, labor market institutions, and employment policies on the transition from school to work and its consequences for the determination of structural unemployment. A second strand of research analyzes the cyclical behavior of youth unemployment and the scarring effects of entering the labor market during recessions.

Starting with the first type of issue, the problems in the school-to-work transition are found on two fronts. The first one is the weak link between education and the labor market. Spain suffers from a high dropout rate—which was especially high during the expansion that preceded the Great Recession—and at the same time, it has a relatively high share of university graduates (mostly in Law, Humanities, and Social Sciences) and a low share of upper secondary education graduates, mostly due to a shortfall in vocational education and training. This peculiar distribution of educational attainment leads to mismatches on the labor market that hamper the labor market integration of youth. The second front is related to labor market institutions, which strongly worsen the employment opportunities of new entrants. With an entrenched dual configuration, both employment protection legislation and the structure of collective bargaining create a strong insider–outsider divide that place a significant burden on youths (Bentolila et al. 2012, 2020; Felgueroso et al. 2018).

Beyond the structural causes of youth unemployment and the long-run trends affecting this segment of the labor market—i.e., demographics, technology, and human capital accumulation—the current cohorts of young workers have been very negatively affected by two deep recessions taking place in short sequence. Indeed, the economic impacts of both the Great Recession and the COVID-19 crisis have been particularly acute in comparison with the rest of Europe. The former was associated with the bursting of a housing bubble and the subsequent downsizing of the construction sector—where many school dropouts were employed—and the latter has strongly reduced employment opportunities in the service sector—in which job entry ports for young people were overrepresented. Thus, we should expect the scars from entering the labor market in a recession to be deeper for the current cohorts of young Spaniards than for their European counterparts. To gauge the magnitude of these adverse effects, we document the impact of previous recessions on the labor market outcomes of youth. Furthermore, for individuals with a college degree, we provide estimates of the career effects of graduating in a recession.

There is a growing literature on the *scarring effects* of recessions. Most studies focus on university graduates and base their empirical strategy on the seminal contribution of Oreopoulos et al. (2012) for the case of Canada. This study exploits the variation across cohorts and provinces in the unemployment rate to study how the labor market conditions at graduation condition entrants’ experience profiles of earnings and employment. In line with Oreopoulos et al. (2012), there are several studies that find substantial scarring effects for college graduates in the US (e.g., Altonji et al. 2016; von Wachter and Schwandt 2019; or Rothstein 2021). According to these studies, deep recessions cause a drop in initial earnings of up to 10%, with estimated semi-elasticities of earnings with respect to the unemployment rate in a range between 2 and 3. The negative effects remain significant up to seven years after entry. The drop in initial earnings is due to the combination of fewer days of work and a reduction in the quality of the entrants’s first employer. Furthermore, the studies for the US and Canada point at substantial variety in the strength of the scarring effects by field of study, college attended, and type of program. In general, the scarring effects are weaker and less persistent for individuals with the highest predicted level of future earnings. Finally, Altonji et al. (2016) and Rothstein (2021) document larger than expected losses for individuals who entered the US labor market during or after the Great Recession. Altonji et al. (2016) attribute this trend break to a rise in the cyclical sensitivity of the employment of college graduates.

Apart from Canada and the US, evidence on the scarring effects of recessions is available for a range of countries.^1^ As for Spain, Fernández-Kranz and Rodríguez-Planas (2018) use Social Security records that cover the period 1980–2008 to analyze the scarring effects from recessions for males at all levels of education, using a setup based on Kahn (2010).^2^ In this simplified setup, the initial unemployment rate is interacted with a continuous variable that measures entrants’ potential experience rather than years of potential experience fixed effects as in Oreopoulos et al. (2012). According to their findings, the negative effects from entry in recessions fall with the educational attainment of entrants. In particular, for high school graduates, they obtain initial earnings losses in deep recessions of 25% that drop to 3% after 10 years. By contrast, for university graduates, the initial impact is half as big and the penalty ceases to be significant after five years.

Our paper offers various contributions to the analysis of youth employment and earnings of young Spaniards. First, our sample period spans the period 1987–2019. Hence, our estimation includes the cohorts that entered during the Great Recession. Furthermore, while our empirical strategy is closer to the one developed by Oreopoulos et al. (2012), we allow for changes over time in the earnings and employment profiles of entrants. This issue is key because inspection of the data reveals a clear negative trend in the initial labor market outcomes of university graduates in Spain. To be more precise, in recessions, we observe a steep deterioration in the initial outcomes of graduates, but in the subsequent recovery, the initial conditions for later cohorts do not recover their pre-recession levels. In other words, a substantial share of the deterioration in labor market outcomes of graduates in recessions is consolidated, causing a trend decline in the initial conditions of university graduates. This result sheds new light on the scarring effects of recessions and casts some doubts about the validity of the standard estimation procedure of Oreopoulos et al. (2012), as it relies on the implicit assumption of a time-invariant relationship between the initial unemployment rate and the experience profiles of entrants.

The rest of this paper is organized as follows. We start by documenting in Sect. 2 the trend deterioration experienced in the labor market for youth in Spain over the last three decades, focusing on aggregate youth unemployment and employment rates, and temporary and part-time employment rates, and we discuss developments in education and in the days worked and earnings of young workers. Then, in Sect. 3, we adopt a cohort approach and show, over the same period, the longitudinal evolution of employment and earnings of young workers from job market entry onwards, finding that losses suffered during recessions are not made up in the subsequent expansion. In Sect. 4, we estimate the size of scarring effects. We trace how the state of the labor market at the graduation dates affects the employment and earnings of young workers during their first decade in the labor market. We find that while there is some evidence of scarring effects, the overriding force appears to be a trend worsening of youth labor market outcomes. In Sect. 5, we summarize the conclusions from our analysis.

## The Spanish youth labor market: an overview

There are well-known reasons why young workers suffer higher unemployment rates and earn lower wages than adult workers. Young workers are more mobile, have lower job experience, and, after transiting from school to work, are more likely to enter into job matches that are of lower duration and less adequate to their professional skills. Typically, as their working life proceeds, workers settle down into significant and stable jobs, where experience, earnings, and job stability improve, resulting in better labor market outcomes.

However, even after taking these factors into account, the Spanish labor market for youth shows a dismal performance.^3^ Three facts are striking. The first one is that differences between the labor market outcomes of young and adult workers are huge in comparison with most European countries. A second related fact is the comparatively slow pace at which Spanish youth enter into more stable and rewarding employment spells. Lastly, a third and less known fact is that the labor market outcomes of Spanish youths have significantly worsened over the last four decades. What follows is a brief overview that documents these three facts.

**Fig. 1:**
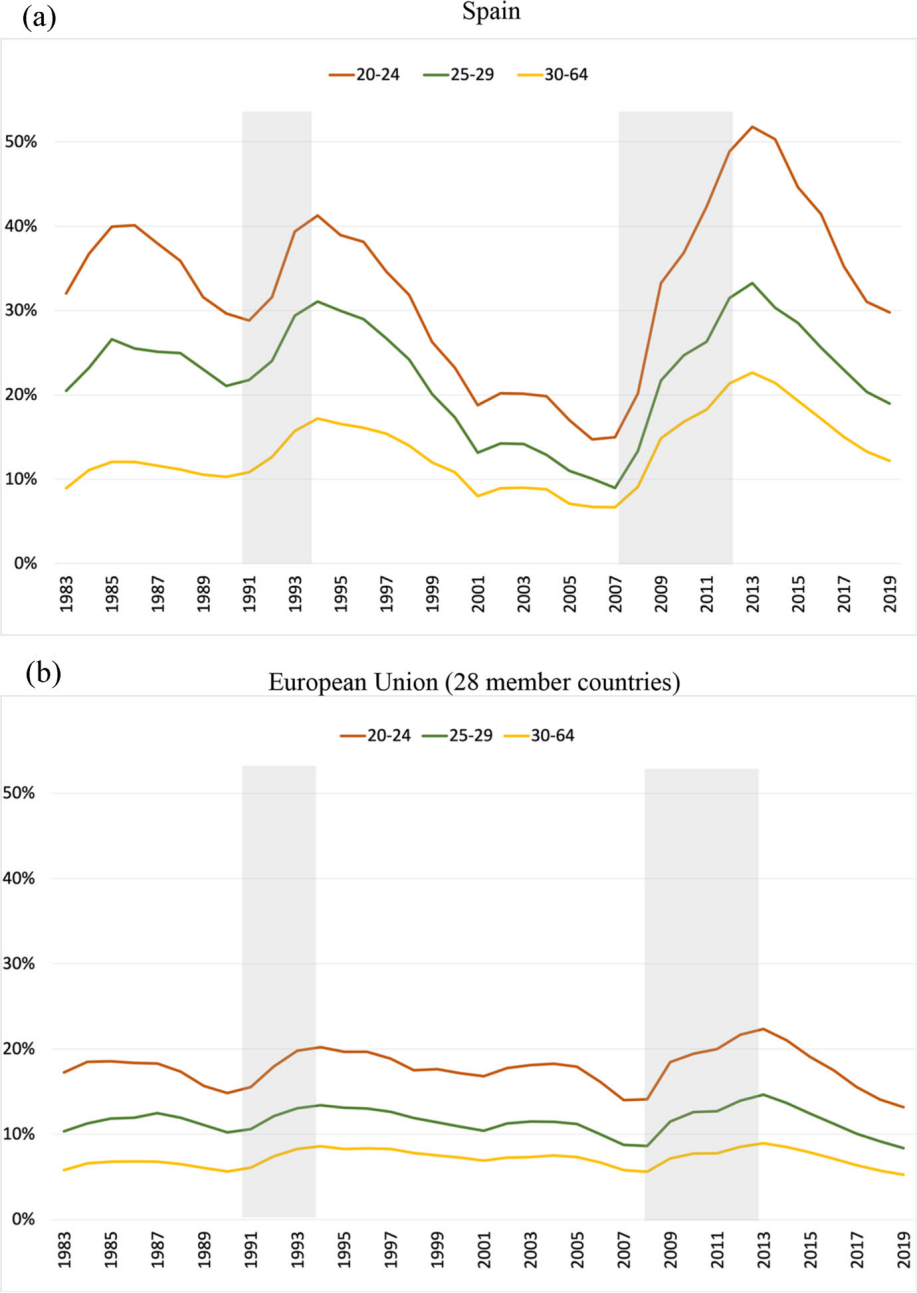
Unemployment rates by age, 1983–2019 (%). **a** Spain; **b** European Union (28 member countries). *Notes*: Annual data. The lines represent the unemployment rates for workers aged 20–24 years old (brown), 25–29 years old (green), and 30–64 years old (orange) (colour figure online). *Source*: OECD Statistics (stats.oecd.org)

To start our discussion, Fig. 1 shows unemployment rates by age group in Spain and the EU-28 from 1983 to 2020. During this period, unemployment rates in Spain fluctuated around an average of 32.7% for the 20–24 year olds, 22.3% for the 25–29 year olds, and 13.1% for the 30–64 year olds.^4^ In contrast, in the EU-28, the corresponding averages were, respectively, 17.8%, 11.5%, and 7.1%. Thus, the gaps between young and adult workers’ average unemployment rates in Spain almost double the ones observed in the EU-28, e.g., 19.6% in Spain versus 10.7% for the 20–24 year olds vis-à-vis the 30–64 year olds.^5^ It is also worth noting that the rises in unemployment rates in recessions (shaded areas in the figure) are much more pronounced in Spain for young workers than for adult workers, while in the EU-28, the unemployment rates for different age groups move more or less in parallel. For instance, while the gap in unemployment rates of the 20–24 year olds with respect to 30–64 year olds in 2007 was the same in the EU-28 and in Spain, 8.2 percentage points (pp), by 2013 it had increased to 13.4 pp in the EU-28 and a whopping 29 pp in Spain.^6^ In the subsequent recovery, the differences between the youth and the adult unemployment rate narrowed substantially, but at the onset of the COVID-19 crisis, the youth unemployment rates were still much higher than at the start of the Great Recession.

**Fig. 2:**
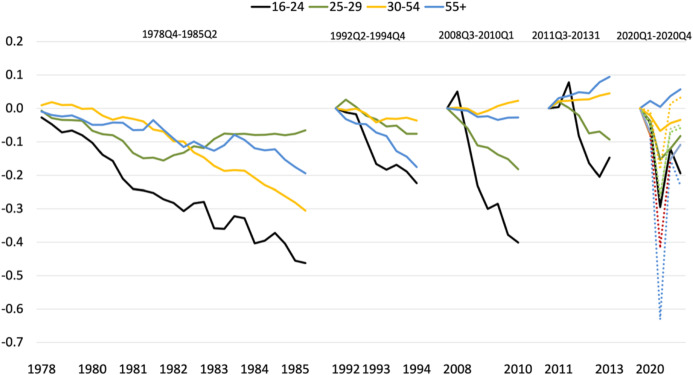
The fall in employment rates in recessions by age group, 1978–2020. *Notes*: The figure shows the difference in log employment rates between the last quarter before the start of a recession and the last quarter prior to the first increase in total employment. The lines correspond to workers aged 16–24 years old (black), 25–29 years old (green), 30–54 years old (orange), and 55 years old and older (blue) (colour figure online). A recession is defined as two consecutive quarters with negative GDP growth rate. For the ongoing COVID-19 recession, we depict all the quarterly variations from 2019Q4 to 2020Q4. Dotted lines depict effective employment excluding temporary layoffs. Sources: National Accounts GDP series and Labor Force Survey, 1976Q3-2020Q4, Instituto Nacional de Estadística (ine.es)

Figure 2 provides a more detailed account of the relative labor market performance of young and adult workers in recent recessions. In each of these recessions, those under 25 suffered the strongest percentage contraction in employment rates, but in the two recessions of the 1980s and early 1990s, young workers in the age group of 25–29 performed better than older age groups. By contrast, the three most recent recessions are characterized by a clear monotonic age pattern with above-average reductions in employment rate for young workers and below-average reductions or even a modest increase in employment rates for prime age workers (30–54 years old) and older cohorts (over 55 years old).

The higher impact of recessions on the employment rates of young workers is a general phenomenon in most countries. Again, there are well-known reasons for this fact. Firms typically use the LIFO principle (last-in, first-out) to adjust their labor force, and employment protection legislation tends to provide higher coverage to older workers (with longer notice periods and higher severance pay, among other provisions). Thus, changes in job flows in recessions typically hit young workers harder. When job creation falls, new entrants to the labor market are especially likely to find fewer and poorer job opportunities. When job destruction increases, those with lower job experience and shorter employment spells are most likely to lose their jobs.

But, again, the magnitude of the differential in employment losses between young and adult workers in Spain is peculiar and it cannot be separated from another characteristic feature of its labor market: the prevalence of temporary contracts in new hires, with a share above 90% since the early 2000s.

**Fig. 3:**
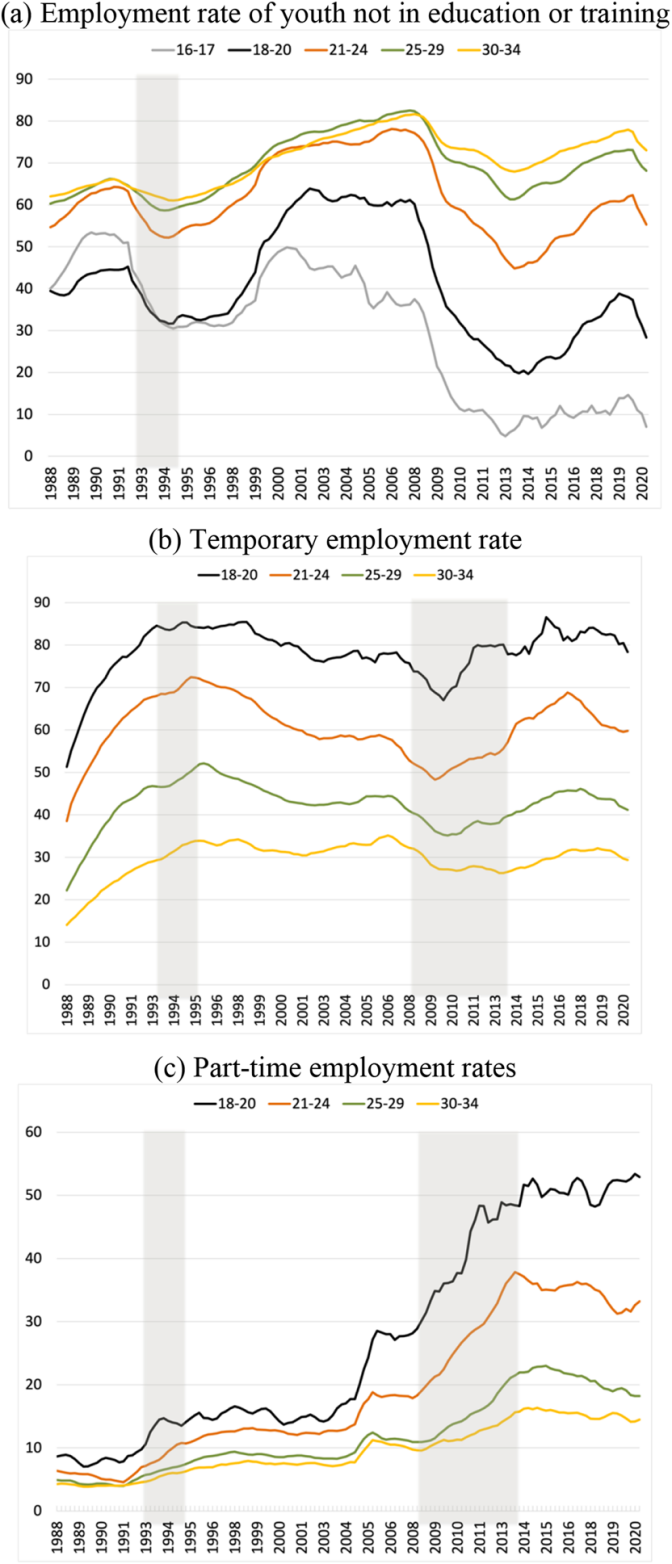
Total, temporary, and part-time employment rates by age, 1988–2020 (%). **a** Employment rate of youth not in education or training. **b** Temporary employment rate. **c** Part-time employment rates. *Notes*: The three panels report four-quarter, backward-looking moving averages. The lines correspond to workers aged 16–17 years old (grey), 18-20 years old (black), 21-24 years old (brown), 25-29 years old (green), and 30-34 years old (black) (colour figure online). *Source*: Labor Force Survey, Instituto Nacional de Estadística (ine.es)

Figure 3 provides information on the employment status of young workers. Panel (a) shows that both the level and the stability of employment rates increase with age. Accordingly, the prevalence of temporary employment falls with age as shown in panel (b): while the average temporary rate has fluctuated between 25 and 33% since the early 1990s (not shown in the figure), for young workers, these rates hover around 80%, 60%, and more than 40%, respectively, for the 18–20, 21–24, and 25–29 year-old groups. Even for employees between 30 and 34 years old, the temporary rate is still around 30%, which suggests that the transition between temporary and regular employment takes place at a very low speed. Lastly, panel (c) shows a marked increase in part-time employment since 2005, which, among other things, is relevant for the evolution of earnings to be described below. The overall picture is very bleak. Before the start of the pandemic, the employment rates for all age groups were still well below their respective peak levels in 2008. Furthermore, the differences in employment rates have grown significantly over time. While the employment rates for individuals above 24 years of age exhibit a positive trend, the employment rates of the youngest age cohorts have plummeted.

The employment rates reported in panel (a) are well below the mean levels in Europe. One factor that contributes to these differences is the slow pace of the school-to-work transition in Spain. Data from the 2009 ad-hoc module of the European Labor Force Survey (LFS) indicated that the average time between leaving formal education and starting the first job for people with upper secondary and post-secondary non-tertiary education was 7.3 months in the EU-27 vis-à-vis 8.8 months in Spain. The figures for people with tertiary education were 5.1 and 7 months, respectively. More recent data indirectly reinforce the idea of a slow transition. In 2019, the employment rate for people aged 15–34 years old in the EU-27 was equal to 75.7%, while in Spain, it was only 64.6%.

The previous graphs are based on repeated cross-sections from the Labor Force Survey. The longitudinal Social Security records from the Continuous Sample of Working Lives (CSWL, see “Appendix 1”) allow a closer examination of the early labor market outcomes of young workers in Spain. For illustrative purposes, we use the 2019 wave of the CSWL to compute annual statistics for earnings and days worked going back to the worker’s time of labor market entry.^7^

**Fig. 4:**
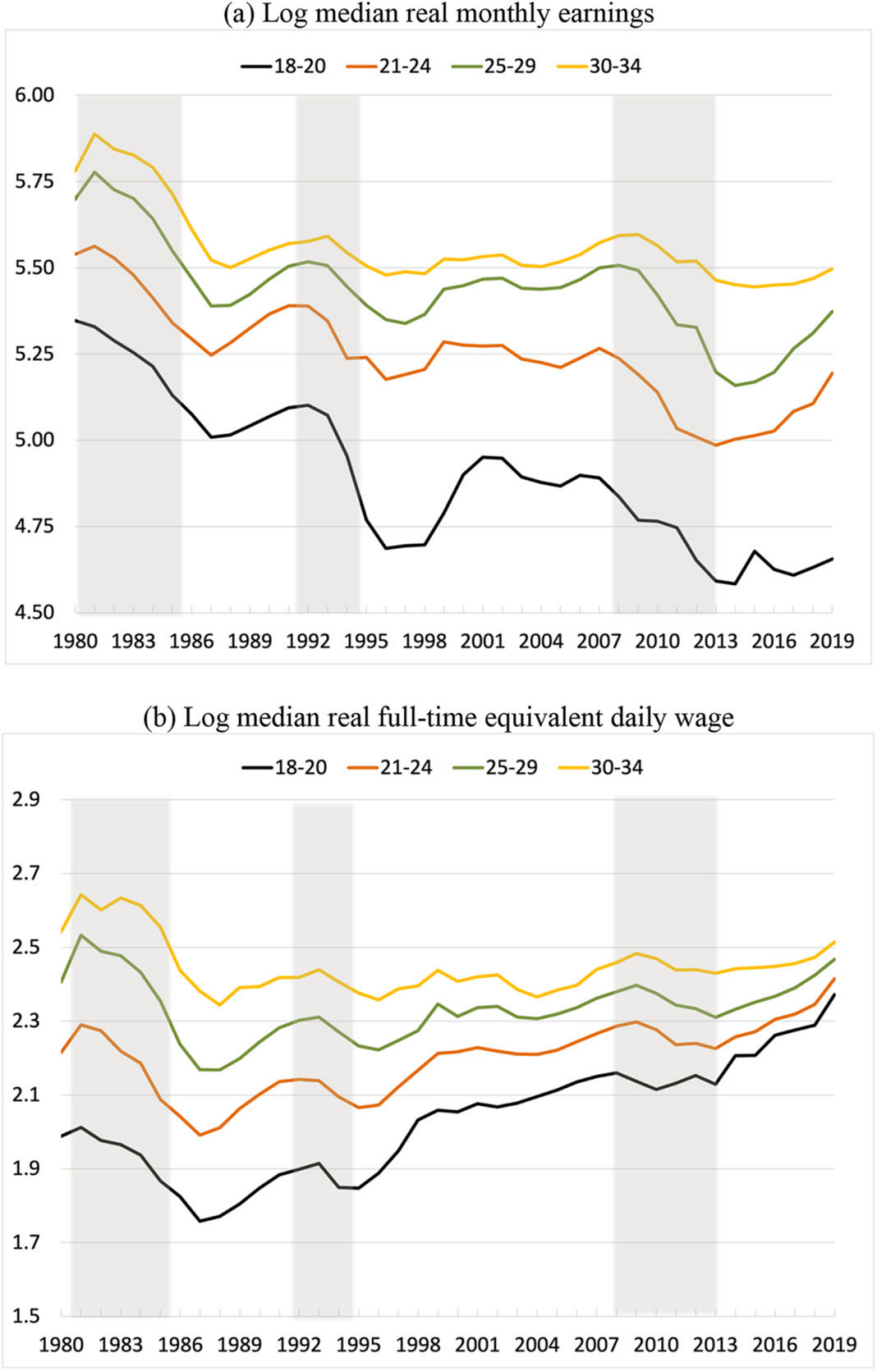

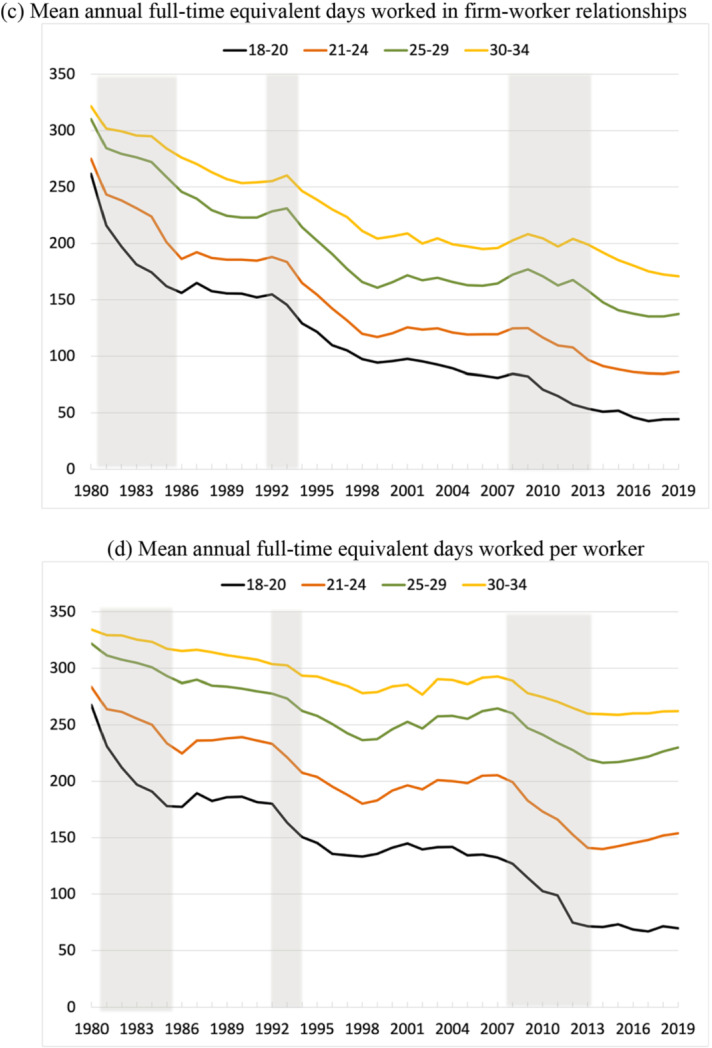
Earnings and annual days worked by age, 1980–2019. **a** Log median real monthly earnings. **b** Log median real full-time equivalent daily wage. **c** Mean annual full-time equivalent days worked in firm-worker relationships. **d** Mean annual full-time equivalent days worked per worker. *Notes*: **a** Log median monthly earnings for employees in work. **b** Log median daily wage converted to full-time equivalent using the part-time coefficient reported in the CSWL. **c** Average length of employment relationships in days in a given calendar year, transformed into full-time equivalent days of work as in **b**. **d** Annual, full-time equivalent days of work for employees and the self-employed working in the corresponding year. The lines correspond to workers aged 18–20 years old (black), 21–24 years old (brown), 25–29 years old (green), and 30–34 years old (orange) (colour figure online). *Source*: Continuous Sample of Working Lives, Ministerio de Inclusión, Seguridad Social y Migraciones

Figure 4 plots the means of four variables: annual days of work of individuals, annual days of work in a given firm-worker relationship, monthly earnings and full-time equivalent daily wages of youths who worked at some point during the year (including self-employment).^8^ For all four age groups, we observe marked negative trends in annual days worked, which are associated to both a reduction in the duration of temporary jobs and the rise of part-time work shown in Fig. 3. As to wages, panel (a) reveals a trend reduction of real monthly earnings, with strong losses during recessions and a partial recovery during expansions. When adjusted for working hours, panel (b) shows the same initial fall, but then sustained rises for all groups subsequently, which are weaker the older is the group of workers and they are still punctuated by reductions during recessions. As we will see below, this increasing pattern for daily wages does not hold when we examine the evolution for university graduates or by cohort. Panels (c) and (d) show that days worked fall sharply in recessions and, surprisingly, barely stabilize in expansions, when job opportunities increase across the board. Indeed, in 2019, our two indicators of the mean days of work were still close to their all-time lows at the end of the Great Recession.

Giving a few figures helps to gauge the magnitudes involved. For the group of workers aged 25–29 years old, real monthly earnings fell from €609 in 1980 to €574 in 2019 (both expressed in 2019 euros), i.e., a 5.7% reduction, while the real daily wage rose from €22.6 to €23.2, namely a 2.5% increase. Over the same period, annual days worked by match for this group fell from 302 to 137, i.e., a 56% reduction, while annual days worked by worker dropped from 322 to 230, namely a 29% decline. Taken together, these numbers imply that the annual average number of jobs per worker increased from 1 to around 1.7. The reductions are even higher for the group of workers aged 21–24 years old: real monthly earnings dropped by 6.2% and the real daily wage grew by 9%, while annual days of work by match fell by 69% and annual days of work by worker were reduced by 46%. These are truly staggering numbers.

It is worth stressing that there are strong composition effects in the evolution of earnings of young workers, since there has been a significant increase in their educational attainment and, consequently, in the age of new entrants to the labor market. Dropout rates among youth have decreased by around 45 pp during the last four decades, while the proportion of university graduates among the young population is now five times larger, in the case of women, and three times larger in the case of males, than in 1980, despite the slowdown observed during the period of the housing bubble (2001–2008).

Consequently, the median age at transiting from school to work has risen by four years for women and by three years for men since the late 1990s. This increase, partly associated to reforms of university education, lowered the employment rates of the youngest workers even more, since simultaneously studying at university and working in either seasonal or part-time jobs is very infrequent in Spain (less than 10% of those aged 18–24 years old do it). Hence, most new entrants to the labor market have no previous labor market experience. Moreover, as shown by Dolado et al. (2000), workers with higher education crowd out less educated ones from their traditional entry jobs, so that a combination of rigid labor market institutions and an increase in the relative supply of workers with higher education harms the training prospects of less educated workers. Furthermore, in a previous version of this paper (Bentolila et al. 2021), we showed that the growth in the supply of university graduates outpaced the growth in demand. Indeed, over the last four decades, the share of university-educated individuals in high-skilled occupations dropped by more than 10 pp.

**Fig. 5:**
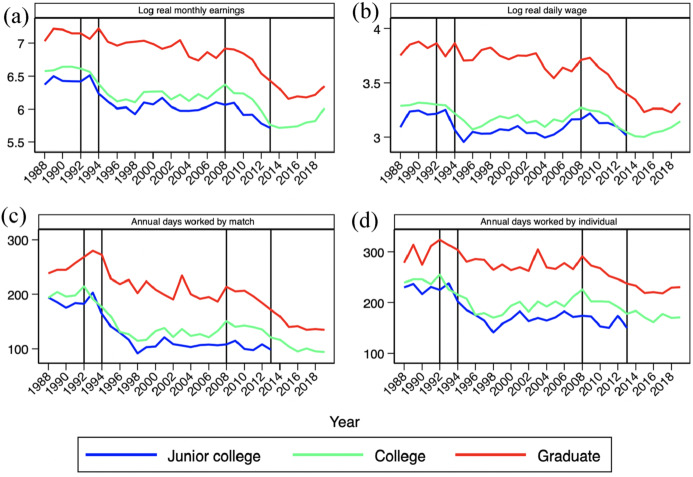
Earnings and annual days worked by new entrants with a university degree, 1988–2019. *Notes*: **a** Log median monthly earnings for employees in work. **b** Log median daily wage converted to full-time equivalent using the part-time coefficient reported in the CSWL. **c** Average length of employment relationships in days in a given calendar year, transformed into full-time equivalent days of work as in **c**. **d** Annual, full-time equivalent days of work for employees and the self-employed working in the corresponding year. The lines correspond to Junior college (blue), College (green), and Graduate studies (red) (colour figure online). *Source*: Continuous Sample of Working Lives, Ministerio de Inclusión, Seguridad Social y Migraciones

Lastly, focusing on new entrants with a university degree, Fig. 5 shows the continuous deterioration of the labor market outcomes for young workers with either junior college (3 years), college (4 years) or graduate degrees.^9^ Again, this arises from strong decreases in annual days worked—either in firm-worker matches or for individual workers during each year—and in wages during recessions, with small gains, if anything, in the subsequent recoveries. Taken together, these evolutions provide a clear signal that there has been a trend worsening of the labor market opportunities for young people. A substantial part of the negative impact of recessions become permanent since it does not get reversed in expansions.

## Youth employment and wages: a descriptive analysis by cohorts

To offer more insight on the deterioration of the labor market outcomes of youth in Spain, we follow different cohorts throughout their working careers from the time of entry to the labor market. In line with our empirical analysis in the next Section, each cohort is defined by their province of residence and year of graduation. The latter is not directly observed but rather imputed from the Labor Force Survey, which allows us to establish a correspondence between the dates of birth and graduation. Similarly, the province of residence at the time of graduation is not observed either. Taking advantage of the low mobility of young Spaniards during their educational period, we take the province of birth as the proxy of the province of residence at the graduation date.^10^

Regarding our outcomes of interest, for each cohort, education group, and calendar year, we examine the same four variables as in Figs. 4 and 5. For worker compensation, we compute the median of each worker’s maximum monthly earnings within the year and maximum daily wage during the year. For employment, we compute the total days worked in each employment spell and the total days worked by each worker throughout the year.^11^ These variables are computed from Social Security administrative data (the CSWL), which capture all employment spells with a daily frequency. All variables except the monthly wage are computed as full-time equivalents using the information provided by the ratio of part-time work (hours worked divided by standard hours). All variables are computed at the cell level. This procedure is standard in the literature, since the seminal article by Oreopoulos et al. (2012).

In what follows, we restrict attention to youth with a university degree. Scarring effects should be more relevant among young people with a university degree, for whom on-the-job human capital accumulation may be severely hampered in recessions. Another reason to focus on university graduates is that their age of entry to the labor market is likely to be less endogenous than for less educated workers. As already indicated, the available data on age at graduation by province allow us to impute the year of labor market entry for these workers. University graduates are split into the three, more homogeneous subgroups we have been considering, i.e., junior college (a minimum of three years), college (initially five years, then reduced to four, see Sect. 4), and graduate studies (master or PhD). This is the maximum degree of disaggregation available in the Social Security data.

**Fig. 6:**
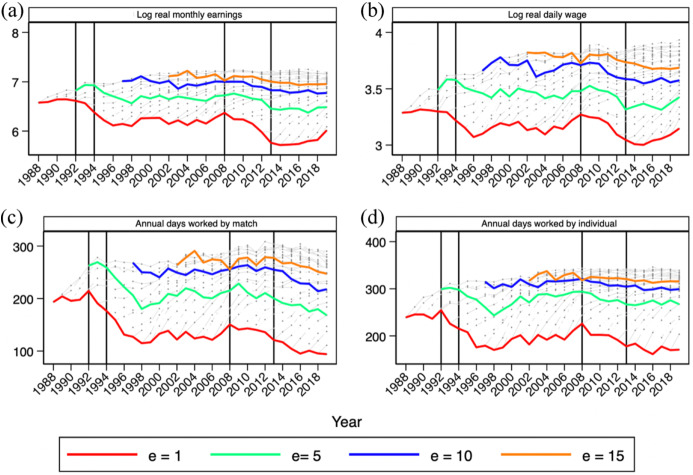
Earnings and employment profiles of new entrants to the labor market with a college degree, 1988–2019. *Notes*: **a** Log median monthly earnings for employees in work. **b** Log median daily wage converted to full-time equivalent using the part-time coefficient reported in the CSWL. **c** Average length of employment relationships in days in a given calendar year, transformed into full-time equivalent days of work as in **c**. **d** Annual, full-time equivalent days of work for employees and the self-employed working in the corresponding year. The levels of these variables are represented for each annual value of potential experience **e**, including lines connecting the values for, respectively, 1 (red), 5 (green), 10 (blue), and 15 years (orange) (colour figure online). *Source*: Continuous Sample of Working Lives, Ministerio de Inclusión, Seguridad Social y Migraciones (color figure online)

Figure 6 plots the results for labor market entrants with a college degree. Apart from the decreasing trend in the entry wage (the red line) already highlighted in Sect. 2, we observe that wages after 5, 10, and 15 years of potential experience (the green, blue, and orange lines, respectively) follow a similar pattern to entry wages. Note that the wage profiles for workers with a graduate degree become flat after 10 years. To some extent this is due to the fact that the CSWL, which is a data set extracted from social security contribution records, only reports capped earnings when the cap for the worker’s contributory base is reached. For this reason, in our analysis of scarring effects in Sect. 4, we stop at 10 years of potential experience.

The figure makes it apparent that earnings losses suffered in recessions are not compensated by higher gains in booms. This is suggestive of the presence of permanent scarring effects. Interestingly, the cohorts entering after 2010 seem to have steeper wage profiles, but in their case, the impact of the ongoing COVID-19 crisis remains to be seen. In the next Section, we provide an assessment of the relative importance of scarring effects and the continuous deterioration of youth labor markets in explaining these patterns.

Table 1 presents the experience profiles by cohort of entry of the four outcomes during the first, fifth, tenth, and fifteenth year of potential experience, i.e., after graduation, over the period 1988–2019. It reveals several features of the data: (a) There are general declines over time, which are also quite large. (b) Monthly earnings fall to a larger extent due to the declines in days of work than to daily wages (though note that this is not a decomposition, given the definitions of the outcomes). (c) The declines in earnings are generally larger the more educated are the workers, while the pattern for annual days worked by worker is more heterogeneous.

**Table 1:** Evolution of labor market outcomes by cohort, education, and experience (%)

Experience	Real monthly earnings	Real daily wage	Annual days worked by match	Annual days worked by worker
*Junior college*				
1	−18.7	1.3	−50.7	−16.9
5	−28.2	−1.6	−32.5	−11.6
10	−16.7	−1.1	−20.3	−8.9
15	−19.0	−6.0	−12.6	−8.4
*College*				
1	−56.4	−14.0	−51.4	−28.6
5	−33.7	−6.6	−36.0	−10.8
10	−23.9	−8.6	−18.8	−5.0
15	−16.0	−13.6	−6.2	−0.5
*Graduate degree*				
1	−68.2	−43.9	−43.2	−17.2
5	−57.0	−30.5	−36.2	−16.1
10	−27.5	−11.5	−18.8	−9.1
15	−16.0	0.6	−3.7	−3.3

The table presents the experience profiles by cohort of entry of the four outcomes during the first, fifth, tenth, and fifteenth year of potential experience over the period 1988–2019. The first two outcomes are log changes and the last two proportional changes

## Estimating scarring effects and trends in youth employment and earnings

### Empirical strategy

Our empirical strategy for estimating the long-term effects of initial labor market conditions on youth employment and earnings exploits the variation in unemployment rates by province of birth and year of graduation but, contrary to the existing literature on scarring effects (see Oreopoulos et al. 2012; Fernández-Kranz and Rodríguez-Planas 2018), we also allow for trend components. These trends are meant to capture any systematic changes that may be present over time in the earnings-experience profiles of labor market entrants.

As already indicated, our outcomes of interest are the log median real monthly earnings, log median real daily wage in full-time equivalents, mean annual days of work in worker-firm matches, and mean annual days of work per worker, distinguishing between three education levels (junior college, college, and graduate degrees). We will also present separate estimates by gender.

Let *y*_*pct*_ denote the relevant labor market outcome in period *t* of a cohort graduated in province *p* in year *c*. Our cell-level reference specification, which is estimated separately for each level of university education, is as follows:

1 $$ y_{pct} = \alpha + \beta_{e} U_{pco} + \theta_{p} + \gamma_{e} +\delta_{e} t +\chi_{c} + \mu U_{t} + \varepsilon_{pct} $$

where *U*_*pc0*_ is the province-level unemployment rate at the time of graduation of cohort *c*, *U*_*t*_ is the aggregate unemployment rate at time *t*, and *θ*_*p*_, *γ*_*e*_, and *χ*_*c*_ represent, respectively, the coefficients of unrestricted province, years of potential experience, and cohort fixed effects. There are 50 provinces, and the sample period is 1987–2019. Only the first ten years of each cohort, when available, are included in the sample.^12^

In this specification, the *β*_*e*_ coefficients capture the persistent effects of the initial conditions at graduation on the labor market outcomes, where the *e* subindex indicates that they are allowed to vary with potential experience (by interacting the unemployment rate with the experience dummies). We include fixed effects for a maximum of ten years of potential experience. Given the presence of province, experience, and cohort fixed effects, the *β*_*e*_ coefficients measure changes in experience profiles in earnings and employment resulting from province-level variations in unemployment at the time of graduation. Notice that we use the overall unemployment rate at the province level and not the youth unemployment rate as the latter variable may be affected by changes in the supply of university graduates.

By choosing the provincial unemployment rate we assume, as most studies in the literature do, that the relevant labor market for generating scarring effects is the local one. Alternatively, if graduates are very mobile geographically, it could be that regional or even national labor market conditions are the sources of scarring effects. As a robustness check, we also estimated specification (1) using the regional unemployment rate (17 regions) instead of the provincial one. As for the national unemployment rate, we use it to control for overall business cycle conditions (coefficient *μ*).^13^ Oreopoulos et al. (2012) include year fixed effects and interact the national unemployment rate with the potential years of experience fixed effects to eliminate lasting effects from the persistence of unemployment rates. By contrast, here we only filter out the common or average contemporaneous impact of the unemployment rate on the labor market outcomes of all cohorts.

The δ_e_ coefficients account for the evolution of labor market outcomes over time during the sample period. As with the scarring effects, we allow time trend effects to differ across individuals with different years of potential experience. Over the more than thirty-year horizon in our sample, there are many factors that may cause structural shifts in experience profiles. On the supply side, Spain has witnessed an enormous rise in the enrollment in university education, especially among women. Therefore, the average quality of university graduates may have changed over time, while the growth in the demand for university graduates lagged behind the growth in its supply in many fields, creating excess supply in the labor market in some fields. Moreover, the introduction of the so-called *Bologna process*, which created the European Higher Education Area, shortened the duration of most college degrees from five to four years and led to a proliferation of new college and graduate degrees. Similarly, on the demand side, skill-biased technological change may have improved the labor market situation of university graduates, but the increased complexity of jobs may also have lengthened their school-to-work transitions. Lastly, institutional factors play a key role in shaping the profiles of entrants. Not only did Spain witness several labor market reforms (in 1994, 1997, 2006, 2010, 2011, and 2012), but these reforms were often implemented in the aftermath of recessions and often contained measures that relaxed the conditions for the use of temporary contracts for young workers. In other words, the adverse conditions during a recession may cause structural shifts in entrant profiles due to the endogenous adoption of reforms.

Finally, the province, years of experience, and cohort fixed effects control for unobserved heterogeneity, due, respectively, to different economic conditions in each local labor market, nonlinear experience profiles, and other different characteristics of the cohorts.^14^

### Identification

Our identification procedure relies on the assumption that the year of graduation and the province of residence are exogenous variables. Selective graduation decisions and self-selection into migration to other provinces (or countries) would undermine our identification. However, notice that we use imputed rather than actual graduation age. This proxy is exogenous from the perspective of an individual and the same is true of our proxy for the province of residence. The province of birth is a powerful predictor of the province of residence at graduation, as almost 90% of Spaniards attend university in their province of birth.^15^ For university graduates in the age group below 35 years old, the share of individuals who live in their province of birth is lower than for all university students. To avoid potential bias in our estimates due to post-graduation migration, as already indicated, we consider a robustness check in which we collapse the individual-level data at the level of cohort, province of first employment after graduation, and calendar year. The results confirm the robustness of our results. Nonetheless, we do not claim that our estimation captures the causal effect of entering the labor market in a recession on the long-term outcomes of young workers. Our method is more akin to a decomposition exercise.

### Data description

The sample period is 1987–2019 for college and graduate workers. The initial date is determined by the availability of individual data in the LFS, while the final date is determined by the availability of the CSWL and the desire to avoid the COVID-19 recession. For junior college workers, it is 1987–2013 since these degrees disappeared after 2013 due to the Bologna process.

Our use of only the 2019 wave of the CSWL implies that the size of the province-cohort-education cells increases over time. For example, for college education, cell size starts at 36 workers in 1987 and it steadily grows up to around 350 in 1996, when it stabilizes.

As to other variables, the average national unemployment rate is 17%, with a standard deviation of 5.3%, ranging from 8.2 to 26.1%. Provincial unemployment rates show a standard deviation of 7.7%, ranging from 3 to 43.2%. The large variation over time and across provinces helps identify the parameters of interest.

Table 2 presents a set of descriptive statistics at the cell level and separately by education level. All variables except for real monthly earnings are measured in full time equivalents. See “Appendix 1” for details about the construction of our sample.

**Table 2:** Descriptive statistics of the cohort-province-year cells

	Mean	SD	P10	P90
*Junior college*				
Real monthly earnings	587.4	217.9	320.2	871.6
Real daily wage	25.8	7.4	18.4	34.1
Annual days worked by match	170.0	68.0	83.2	255.3
Annual days worked by worker	228.4	71.5	130.2	311.3
*College*				
Real monthly earnings	687.8	257.4	345.2	1031.0
Real daily wage	29.2	8.0	19.9	38.7
Annual days worked by match	184.5	64.3	99.4	261.7
Annual days worked by worker	246.1	65.3	154.1	318.6
*Graduate*				
Real monthly earnings	984.1	355.3	486.9	1381.6
Real daily wage	40.7	35.1	25.0	49.3
Annual days worked by match	219.3	80.5	121.9	365.0
Annual days worked by worker	279.8	74.2	190.4	365.0

Period: 1987–2019. The cell sizes are 8809 for Junior college, 9929 for College, and 7134 for Graduate degree. The unit of analysis is the cohort-province-year cell. P10 denotes the first decile and P90 the ninth decile. All variables except for real monthly earnings are measured in full time equivalents. Monthly earnings and wages are expressed in real euros with 1987 as the base year. *Source*: Continuous Sample of Working Lives, Ministerio de Inclusión, Seguridad Social y Migraciones

The table shows that both earnings and days of work increase with education, as expected, though the slope between junior college and college is not very pronounced (with an increase in 17% in monthly earnings). The level of monthly earnings is quite low, showing extremely low values at the first decile and the ninth decile, though they are not adjusted for days or hours of work. Annual days of work by match are also very low, with the averages going from 5.6 to 7.2 months, which reflects the prevalence of very short temporary contracts. Annual days by worker are also relatively low, ranging from 7.5 to 9.2 months per year. The difference between the two variables indicates that young workers quickly move from one temporary contract to another.

### Baseline results

We now report the estimates of our reference specification (Eq. 1) and compare them to more restrictive alternative specifications that do not fully allow for scarring, trend, and business cycle effects, and unobserved heterogeneity. To streamline the presentation of the results, we will subsequently present the results for the following three specifications:

- *Model 1* includes province and years of potential experience fixed effects, as well as interactions of the latter with the initial province-level unemployment rate at graduation, which capture the scarring effects at different stages of working lives.^16^
- *Model 2* includes province and years of potential experience fixed effects, scarring effects (as in Model 1), and adds linear trends interacted with years of potential experience, which can capture a secular decline in youth labor market outcomes at different stages of working lives.
- *Model 3* is our reference specification, Eq. (1), which includes scarring, trend, and business cycle effects, and province, years of potential experience, and cohort fixed effects.

**Fig. 7:**
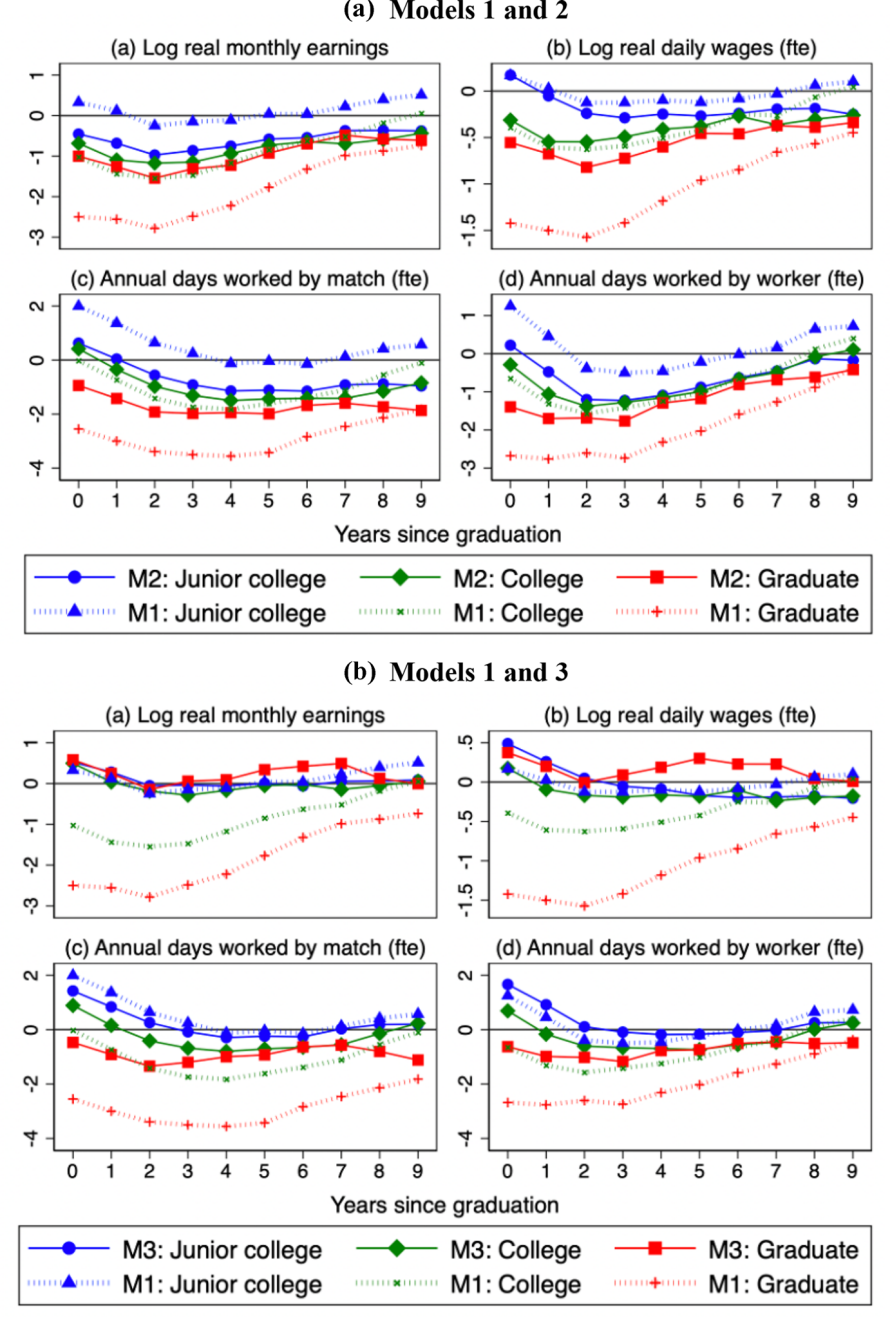
Estimated scarring effects in Models 1–3. **a** Models 1 and 2; **b** Models 1 and 3. *Notes*: The figure shows the scarring effects estimated for the three education groups in Models 1–3 (Tables 3, 4, 5), namely the coefficients on the provincial unemployment rate in the year of graduation, interacted with fixed effects for the years of potential experience, from the first (0) to the tenth (9). The symbols correspond to: (1) Model 1: Junior college (blue triangle), College (green x sign), and Graduate studies (red plus sign). (2) Models 2 and 3: Junior college (blue circle), College (green diamond), and Graduate studies (red square) (colour figure online). The coefficients for monthly earnings and daily wages are multiplied by 100. All outcomes except for earnings are full time equivalents (fte)

Tables 3, 4, and 5 report, respectively, the main results from the estimation of Models 1, 2, and 3. They present only the estimated scarring and trend effects on entry (labeled year 0) and in the fifth (year 4) and tenth year (year 9), while the full results are included in the Online Appendix. Standard errors are clustered at the graduation year-province level. Figure 7 shows the scarring effects estimated under the three specifications. Since we consider three education groups, four outcome variables, and several empirical specifications, we will focus on the estimates for college graduates, who are by far the largest group. The results for the other two education levels are qualitatively similar, so we will just briefly comment on them for Model 3.

**Table 3:** Estimates of scarring effects: model 1

	Log real monthly earnings			Log real daily wage		
	Junior college	College	Graduate	Junior college	College	Graduate
U0 x (*e* = 0)	0.003	−0.010***	−0.025***	0.002	−0.004***	−0.014***
	(0.002)	(0.002)	(0.003)	(0.001)	(0.001)	(0.002)
U0 x (*e* = 4)	−0.001	−0.012***	−0.022***	−0.001	−0.005***	−0.012***
	(0.001)	(0.001)	(0.002)	(0.001)	(0.001)	(0.001)
U0 x (*e* = 9)	0.005***	0.001	− 0.007***	0.001	0.000	−0.040***
	(0.001)	(0.001)	(0.002)	(0.001)	(0.001)	(0.001)
Constant	5.902***	6.037***	6.786***	3.980***	3.183***	3.605***
	(0.050)	(0.047)	(0.087)	(0.024)	(0.020)	(0.052)
Observations	8809	9929	7134	8808	9929	7132
*R*^2^	0.535	0.689	0.457	0.520	0.716	0.444

	Annual days worked by match			Annual days worked by worker		
	Junior college	College	Graduate	Junior college	College	Graduate
U0 x (*e* = 0)	1.996***	−0.020	−2.550***	1.251***	−0.647***	−2.679***
	(0.288)	(0.207)	(0.314)	(0.314)	(0.207)	(0.275)
U0 x (*e* = 4)	−0.124	−1.824***	−3.558***	−0.461**	−1.240***	−2.316***
	(0.216)	(0.163)	(0.257)	(0.195)	(0.140)	(0.208)
U0 x (*e* = 9)	0.572***	−0.100	−1.819***	0.724***	0.406***	−0.373
	(0.193)	(0.182)	(0.442)	(0.156)	(0.131)	(0.335)
Constant	71.138***	82.539***	205.413***	127.772***	150.286***	282.182***
	(8.216)	(6.803)	(11.329)	(8.288)	(6.048)	(11.334)
Observations	8809	9929	7134	8809	9929	7134
*R*^2^	0.524	0.662	0.377	0.636	0.764	0.399

The sample period is 1987–2019 for college and graduate degrees. For junior college workers it is 1987–2013. The model includes fixed effects for the province of birth and years elapsed since graduation. Standard errors clustered at the graduation year-province level are in parentheses. ***p < 0.01; **p < 0.05; *p < 0.10

**Table 4:** Estimates of scarring and trend effects: model 2

	Log real monthly earnings			Log real daily wage		
	Junior college	College	Graduate	Junior college	College	Graduate
U0 x (*e* = 0)	−0.005**	−0.007***	−0.010***	0.002	−0.003***	−0.006***
	(0.002)	(0.001)	(0.002)	(0.001)	(0.001)	(0.001)
U0 x (e = 4)	−0.008***	−0.009***	−0.012***	−0.002***	−0.004***	−0.006***
	(0.001)	(0.001)	(0.001)	(0.001)	(0.001)	(0.001)
U0 x (e = 9)	−0.004***	−0.004***	−0.006***	−0.002**	−0.003***	−0.003***
	(0.001)	(0.001)	(0.002)	(0.001)	(0.001)	(0.001)
Trend x (e = 0)	−0.024***	−0.0230***	−0.048***	0.004***	−0.007***	−0.027***
	(0.002)	(0.001)	(0.002)	(0.001)	(0.001)	(0.002)
Trend x (*e* = 4)	−0.017***	−0.013***	−0.022***	−0.004***	−0.006***	−0.013***
	(0.001)	(0.001)	(0.001)	(0.001)	(0.001)	(0.001)
Trend x (*e* = 9)	−0.016***	−0.013***	−0.012***	−0.007***	−0.008***	−0.005***
	(0.001)	(0.001)	(0.001)	(0.001)	(0.001)	(0.001)
Constant	6.361***	6.444***	7.725***	3.037***	3.301***	4.143***
	(0.061)	(0.043)	(0.078)	(0.034)	(0.023)	(0.047)
Observations	8809	9929	7134	8808	9929	7132
*R*^2^	0.641	0.799	0.655	0.538	0.755	0.625

	Annual days worked by match			Annual days worked by worker		
	Junior college	College	Graduate	Junior college	College	Graduate
U0 x (*e* = 0)	0.628**	0.416**	−0.940***	0.223	−0.288	−1.393***
	(0.267)	(0.185)	(0.238)	(0.307)	(0.21)	(0.237)
U0 x (*e* = 4)	−1.143***	−1.496***	−1.947***	−1.089***	−1.150***	−1.291***
	(0.208)	(0.184)	(0.213)	(0.188)	(0.163)	(0.199)
U0 x (*e* = 9)	−0.960***	−0.841***	−1.865***	−0.171	0.107	−0.417
	(0.195)	(0.183)	(0.322)	(0.180)	(0.149)	(0.272)
Trend x (*e* = 0)	−4.196***	−3.044***	−4.924***	−3.464***	−2.332***	−4.135***
	(0.372)	(0.157)	(0.290)	(0.364)	(0.164)	(0.250)
Trend x (*e* = 4)	−2.557***	−1.939***	−3.863***	−1.549***	−0.534***	−2.446***
	(0.213)	(0.177)	(0.253)	(0.182)	(0.143)	(0.219)
Trend x (*e* = 9)	−2.700***	−1.861***	−3.118***	−1.515***	−0.768***	−2.139***
	(0.223)	(0.184)	(0.335)	(0.182)	(0.133)	(0.290)
Constant	152.74***	136.86***	303.72***	194.13***	191.51***	364.10***
	(11.699)	(6.908)	(10.410)	(11.236)	(6.638)	(10.799)
Observations	8809	9929	7134	8809	9929	7134
*R*^2^	0.634	0.755	0.545	0.682	0.785	0.509

The sample period is 1987–2019 for college and graduate degrees. For junior college workers, it is 1987–2013. The model includes fixed effects for the province of birth and years elapsed since graduation. Standard errors clustered at the graduation year-province level are in parentheses. ***p < 0.01; **p < 0.05; *p < 0.10

**Table 5:** Estimates of scarring and trend effects: model 3

	Log real monthly earnings			Log real daily wage		
	Junior college	College	Graduate	Junior college	College	Graduate
U0 x (*e* = 0)	0.005**	0.005***	0.006*	0.005***	0.002	0.004*
	(0.002)	(0.002)	(0.003)	(0.002)	(0.001)	(0.002)
U0 x (*e* = 4)	−0.001	−0.002	0.001	−0.001	−0.002	0.002
	(0.002)	(0.001)	(0.003)	(0.002)	(0.001)	(0.002)
U0 x (*e* = = 9)	0.001	0.001	0.001	−0.002	−0.002	0.001
	(0.002)	(0.002)	(0.003)	(0.001)	(0.001)	(0.002)
Trend x (*e* = 0)	−0.0270***	−0.016***	−0.035***	0.002	−0.005***	−0.019***
	(0.002)	(0.003)	(0.003)	(0.002)	(0.002)	(0.002)
Trend x (*e* = 4)	−0.019***	−0.005*	−0.012***	−0.005***	−0.003*	−0.007***
	(0.002)	(0.003)	(0.003)	(0.002)	(0.002)	(0.002)
Trend x (*e* = 9)	−0.015***	−0.003	−0.013***	−0.007***	−0.004**	−0.006**
	(0.002)	(0.003)	(0.004)	(0.002)	(0.002)	(0.002)
National UR	−0.008***	−0.007***	−0.007***	−0.005***	−0.004***	−0.004***
	(0.001)	(0.001)	(0.001)	(0.001)	(0.001)	(0.001)
Constant	6.484***	6.498***	7.342***	3.148***	3.357***	3.907***
	(0.067)	(0.054)	(0.107)	(0.042)	(0.033)	(0.070)
Observations	8809	9929	7134	8808	9929	7132
*R*^2^	0.665	0.812	0.687	0.561	0.766	0.656

	Annual days worked by match			Annual days worked by worker		
	Junior college	College	Graduate	Junior college	College	Graduate
U0 x (*e* = 0)	1.425***	0.891***	−0.470	1.665***	0.692***	−0.627
	(0.348)	(0.233)	(0.420)	(0.366)	(0.238)	(0.397)
U0 x (*e* = 4)	−0.292	−0.798***	−0.997**	−0.179	−0.695***	−0.768**
	(0.278)	(0.209)	(0.401)	(0.279)	(0.188)	(0.374)
U0 x (*e* = 9)	0.200	0.235	−1.118**	0.263	0.245	−0.482
	(0.283)	(0.241)	(0.468)	(0.284)	(0.209)	(0.464)
Trend x (*e* = 0)	−4.520***	−3.032***	−4.391***	−4.174***	−2.087***	−3.945***
	(0.431)	(0.422)	(0.417)	(0.431)	(0.790)	(0.519)
Trend x (*e* = 4)	−2.940***	−2.018***	−3.831***	−1.845***	−0.483	−2.669***
	(0.372)	(0.421)	(0.427)	(0.368)	(0.790)	(0.526)
Trend x (9)	−2.777***	−1.958***	−4.369***	−1.450***	−0.449	−3.096***
	(0.443)	(0.485)	(0.488)	(0.432)	(0.819)	(0.589)
National UR	0.101	0.387***	0.552***	−1.518***	−1.181***	−0.742***
	(0.155)	(0.126)	(0.165)	(0.144)	(0.122)	(0.143)
Constant	161.72***	146.40***	269.72***	224.51***	218.16***	356.84***
	(12.145)	(8.191)	(13.668)	(11.999)	(7.545)	(14.615)
Observations	8809	9929	7134	8809	9929	7134
*R*^2^	0.659	0.777	0.576	0.705	0.806	0.525

The sample period is 1987–2019 for college and graduate degrees. For junior college workers, it is 1987–2013. The model includes fixed effects for the province of birth, years elapsed since graduation, and graduation cohort. National UR denotes the national unemployment rate. Standard errors clustered at the graduation year-province level are in parentheses. ***p < 0.01; **p < 0.05; *p < 0.10

The estimated coefficients for Model 1 (see Table 3 and Fig. 7a) clearly suggest that the initial conditions faced at graduation have a strong and persistent impact on entrant earnings. For instance, for monthly earnings, the semi-elasticity of with respect to the unemployment rate at the time of graduation reaches a maximum of − 1.5 in the second year and it becomes − 0.5 seven years after graduation, losing significance thereafter. The coefficients in the graph are multiplied by 100, so this estimate means that an increase of 1 pp in the provincial unemployment rate is associated with a drop in monthly earnings of 1.5%. To get a sense of the magnitudes, this implies that over the Great Recession, a young person entering the labor market in 2013 in the province with the lowest unemployment rate in 2007 (Guipúzcoa) would have 13.5% lower monthly earnings two years later than an entrant in 2007, while the corresponding figure for the province with the largest unemployment rate (Jaén) would be a staggering 39.4% loss. The impact of unemployment on the full-time equivalent daily wage in this specification is lower, reaching a maximum of − 0.5 in year 2 and dropping to − 0.3 in year 9, which reveals that part of the effect on monthly earnings comes from lower hours of work. The impact of a 1 pp increase in the provincial unemployment rate on annual days worked by worker in year 2 is equal to 1.6 days less and to 1.4 less days worked by match (climbing to 1.8 days in year 4).

These estimates are smaller than those for the annual earnings of college graduates in the related literature. In particular, for the US Oreopoulos et al. (2012, Table 2) estimates the peak effect is − 1.8% in years 0–1, even though their specification includes fixed effects by region of residence, graduation cohort, potential labor market experience, and calendar year.

For Spain, Fernández-Kranz and Rodríguez-Planas (2018, Table 2) estimate a peak effect of − 1.6% in the year of entry, including the same fixed effects as Oreopoulos et al. (2012) except that they replace the experience fixed effects by a linear and a quadratic trend. Given this different specification, our results are not directly comparable with theirs. Moreover, they estimate the effects on annual earnings, while we examine maximum monthly earnings, which we see as more appropriate to deal with the top coding that is present in the data, and our period includes the Great Recession whereas their period does not.

Estimated scarring effects are significantly lower in Model 2 (see Table 4 and Fig. 7a), which includes the interacted linear trends to control for changes in the conditions of youth labor markets. In some instances, the precision of the estimates increases. The semi-elasticity of the monthly earnings of college graduates to initial unemployment attains a maximum of − 1.2 in year 2 and it is equal to − 0.5 for daily earnings. Similarly, the impact on annual days worked by match reaches a peak of 1.5 days less in year 4 and to 1.4 days less in year 2 on annual days by worker. These effects have a relatively small magnitude. Thus, while scarring effects are often mentioned as the main culprits of the worsening of the labor market outcomes of young Spaniards, these results suggest that there are also other causes of the deterioration of young workers’ wages and employment beyond the state of the labor market at the time of graduation. And this is despite there being two deep recessions in our sample period (1992–1994 and 2008–2013).

The estimated coefficients of the linear trends indicate a continuous and sizeable deterioration in the earnings of young workers upon entry; being equal to − 2.3% per year for monthly earnings and − 0.7% for full-time equivalent daily wage. The corresponding figures are 3 days worked less per match and 2.3 days worked less per worker. To give a sense of magnitudes, if we compare two similar workers entering the labor market ten years apart, the worker with the later entry would have a 7% lower daily wage and would work 23 days less, i.e., a 9.3% reduction with respect to the average days worked shown in Table 2. These are very large impacts that swamp the estimated scarring effects.

Moving now to our reference specification, Model 3 (see Table 5 and Fig. 7b), the estimated scarring effects again change significantly. For college workers, scarring effects on monthly earnings and daily wages essentially vanish (showing an anomalous small, but positive effect in the first year), while they remain significant for annual days of work. At the peak in year 4, a 1 pp increase in the provincial rate of unemployment on entry reduces days worked per match by 0.8 days and days worked per worker by 0.7 days (see the Online Appendix).

The trend deterioration is sizeable. Taking college-educated workers, the effect on entry is equal to 1.6% lower monthly earnings and 0.5% daily wages, and 3 days less by match per year and 2.1 days less by worker per year. Therefore, in their year of entry to the labor market, a worker entering 10 years later than another worker with similar characteristics would earn 16% less in his best month of the year and 5% less in his best-earning day of the year, and they would stay one month less per match and work 3 weeks less per year. These trends are larger in the first few years in a person’s working life, which suggests the presence of increasing problems in the school-to-work transition of Spanish youth.

It is interesting to compute the cumulative impact of the estimated effects on monthly earnings and annual days by worker. To this aim, we add up the coefficients on the yearly estimates from Model 3 over a ten-year horizon, considering only those coefficients which are significant at the 10% level, and use an annual discount rate of 3%. There are no scarring effects on earnings, whereas in the case of annual days of work by worker, the cumulated scarring effect amounts to 2.7 days less for each point of increase in the provincial unemployment rate. On the other hand, the cumulated trend effect reduces monthly earnings by 5.6% and by 3.4 days worked less with every new year.

These cumulated effects are smaller than in Model 1, which does not include the trends, the cohort fixed effects or the national unemployment rate. For monthly earnings in that model, the cumulated effect adds up to a drop of 8.1% per extra point increase in provincial unemployment, vis-à-vis 5.6% in the full model. For annual days of work by worker, the estimated total is equal to 7.4 days less per year extra year of entry in the labor market, as opposed to 2.7 days in Model 3.

Turning now to the impact of the current national unemployment rate, it is negative and statistically significant, with a semi-elasticity of − 0.7, i.e., a 1 pp increase in the national unemployment rate reduces real monthly earnings by 0.7%, while the effect on the full-time equivalent daily wage is 0.4%. Over the Great Recession, the national unemployment rate increased by roughly 18 pp, from 8.2% in 2007 to 26.1% in 2013. Our estimate implies that the daily wage of a worker entering the labor market at the end of the Great Recession in 2013 would be 7.2% lower than a similar worker entering in 2007. Let us also note that the unemployment rate has a positive impact (of 0.4 days) on days worked by match, which turns negative (at 1.2 days less) for days worked by worker. This suggests that when the labor market worsens firms destroy the matches with the lowest duration (and most likely on temporary contracts). Finally, the inclusion of cohort unobserved heterogeneity and the aggregate unemployment rate does not make the statistical significance of the negative linear trends go away.

Since the move from Model 2 to Model 3 involves two steps, it is worth checking the extent to which each of them is responsible for the fall in the estimated scarring effects. To this effect, Table 8 in presents the estimates for college graduates for Models 1–3, including a model (labeled Model 3a) which adds to Model 2 only the cohort fixed effects, revealing that the scarring effects lose significance as soon as these effects are included in the regression.

It is also worth noting that according to the estimates of Model 3, workers with different education levels suffer different impacts. First, scarring effects still increase with the level of education at the peak for days worked, while this is no longer the case for earnings or wages, since they are not significant. Second, the trend deterioration of all four labor market outcomes shows a U-shape with education, being largest for workers with a junior college degree, then for graduate degrees, and smallest for workers with a college degree (see the Online Appendix).

Uncovering the factors that generate the continuous deterioration of the youth labor market conditions that we capture with the linear trends would require explicitly measuring the factors underlying the unobserved cohort heterogeneity and time effects, including those related to institutional changes, such as employment policies, labor market regulation, etc. We leave this task for future work. For now, our results call into question the magnitude of the scarring effects that have been mentioned to explain the worsening of the labor market outcomes of Spanish young workers.

### Robustness checks

Regarding observed cohort heterogeneity, there are several dimensions in which the size and composition of the cohorts may differ, but in our view, the most important one is the gender composition. This is true for three reasons: (a) there are gender gaps in wages and employment rates among young Spaniards, which have been changing over time, (b) the weight of women in the cohorts entering the labor market has increased substantially during the sample period, and (c) women tend to be overrepresented in fields of study with worse labor market prospects.

Hence, we also estimated wage and employment equations separately for men and women. Table 6 provides the main results by reporting scarring effects and cohort trends at the year of graduation (*e* = 0) and 5 and 9 years since graduation, for college graduates and for the three empirical specifications discussed above. Overall, we find similar scarring effects across gender groups and more negative time trends in the case of men, although the scarring effects mostly disappear when we control for the trends and the national unemployment rate. In any case, our key conclusions remain: the worsening of labor market outcomes of young Spaniards result from a combination of some scarring effects from local conditions at the time of graduation and, especially, a continuous deterioration of their wages and employment rates not only at the time of transiting from school to work, but also some years after graduation.

**Table 6:** Estimates of scarring and trend effects for college education by gender: models 1–3

	Real monthly earnings		Real daily wage		Days worked by match		Days worked by worker	
	Men	Women	Men	Women	Men	Women	Men	Women
Model 1. Interactions of initial unemployment with experience								
U0 x (*e* = 0)	−0.004***	−0.005***	−0.002***	−0.002***	−0.144	−0.089	−0.333***	−0.379***
	(0.001)	(0.001)	(0.001)	(0.001)	(0.125)	(0.121)	(0.125)	(0.121)
U0 x (*e* = 4)	−0.003***	−0.005***	−0.002***	−0.002***	−0.700***	−0.876***	−0.446***	−0.628***
	(0.001)	(0.001)	(0.001)	(0.001)	(0.107)	(0.091)	(0.074)	(0.083)
U0 x (*e* = 9)	0.001*	0.001	0.001***	0.001	0.027	0.079	0.161**	0.163**
	(0.001)	(0.001)	(0.001)	(0.001)	(0.108)	(0.108)	(0.066)	(0.077)
Constant	6.21***	5.92***	3.22***	3.18***	120.55***	79.51***	187.48***	142.85***
	(0.049)	(0.074)	(0.029)	(0.043)	(6.839)	(8.984)	(6.885)	(10.225)
Observations	9458	9596	9457	9595	9459	9596	9459	9596
*R*^2^	0.574	0.616	0.587	0.605	0.513	0.583	0.619	0.674
Model 2. Interactions of initial unemployment with experience and trends								
U0 x (*e* = 0)	−0.003***	−0.002**	−0.002***	−0.001***	−0.022	0.256**	−0.240**	−0.113
	(0.001)	(0.001)	(0.001)	(0.001)	(0.103)	(0.113)	(0.119)	(0.125)
U0 x (*e* = 4)	−0.002***	−0.004***	−0.001***	−0.002***	−0.558***	−0.684***	−0.402***	−0.595***
	(0.001)	(0.001)	(0.001)	(0.001)	(0.096)	(0.102)	(0.079)	(0.094)
U0 x (*e* = 9)	0.001	0.001	0.001	0.001	−0.130	−0.155	0.097	0.099
	(0.001)	(0.001)	(0.001)	(0.001)	(0.114)	(0.109)	(0.074)	(0.087)
Trend x (*e* = 0)	−0.024***	−0.022***	−0.010***	−0.004***	−2.669***	−3.234***	−1.940***	−2.430***
	(0.001)	(0.002)	(0.001)	(0.001)	(0.194)	(0.194)	(0.216)	(0.211)
Trend x (*e* = 4)	−0.014***	−0.012***	−0.008***	−0.003***	−1.764***	−1.909***	−0.503***	−0.296*
	(0.001)	(0.001)	(0.001)	(0.001)	(0.193)	(0.201)	(0.152)	(0.175)
Trend x (*e* = 9)	−0.011***	−0.008***	−0.006***	−0.003***	−10.077***	−1.530***	−0.463***	−0.453**
	(0.001)	(0.001)	(0.001)	(0.001)	(0.242)	(0.226)	(0.165)	(0.188)
Constant	6.67***	6.31***	3.40***	3.24***	170.29***	136.54***	223.26***	185.51***
	(0.054)	(0.067)	(0.035)	(0.045)	−8.132	−8.511	−8.063	− 10.534
Observations	9458	9596	9457	9595	9459	9596	9459	9596
*R*^2^	0.672	0.699	0.639	0.618	0.578	0.670	0.636	.695
Model 3. Interactions of initial unemployment with experience and trends, cohort fixed effects, national unemployment								
U0 x (*e* = 0)	0.001	0.002**	0.001	0.001	0.062	.506***	0.064	0.419***
	(0.001)	(0.001)	(0.001)	(0.001)	(0.123)	(0.117)	(0.137)	(0.127)
U0 x (*e* = 4)	0.001	−0.001**	0.001	−0.001*	−0.300***	−0.314***	−0.264***	−0.308***
	(0.001)	(0.001)	(0.001)	(0.001)	(0.107)	(0.095)	(0.093)	(0.087)
U0 x (*e* = 9)	0.001	0.002***	0.001	0.001	0.253*	0.386***	0.123	0.241**
	(0.001)	(0.001)	(0.001)	(0.001)	(0.131)	(0.125)	(0.091)	(0.107)
Trend x (*e* = 0)	−0.019***	−0.011***	−0.009***	− 0.003**	−2.861***	−2.781***	−1.879***	−1.497**
	(0.002)	(0.003)	(0.001)	(0.001)	(0.339)	(0.406)	(0.500)	(0.660)
Trend x (*e* = 4)	−0.008***	0.001	−0.008***	−0.001	−20.03***	−1.509***	−0.533	0.506
	(0.002)	(0.003)	(0.002)	(0.001)	(0.352)	(0.412)	(0.507)	(0.662)
Trend x (*e* = 9)	−0.008***	0.006*	−0.007***	0.001	−1.916***	−1.375***	−0.363	0.394
	(0.002)	(0.003)	(0.002)	(0.001)	(0.443)	(0.472)	(0.539)	(0.704)
National UR	−0.006***	−0.005***	−0.004***	−0.002***	0.735***	−0.91***	−10.06***	
	(0.001)	(0.001)	(0.001)	(0.001)	(0.154)	(0.136)	(0.133)	(0.120)
Constant	6.71***	6.43***	3.45***	3.32***	172.56***	145.19***	247.90***	208.13***
	(0.064)	(0.069)	(0.045)	(0.048)	−9.561	−9.942	−9.179	−10.623
Observations	9458	9596	9457	9595	9459	9596	9459	9596
*R*^2^	0.693	0.72	0.656	0.634	0.608	0.692	0.655	0.714

The sample period is 1987–2019. Models 1 and 2 include fixed effects for the province of birth and years elapsed since graduation. Model 3 adds graduation cohort. Standard errors are in parentheses. ***p < 0.01; **p < 0.05; *p < 0.10

Two other robustness checks are presented in Table 7. The first column shows the estimates of Model 3 for reference. The second column uses the province of the workers’ first job instead of their province of birth. The estimates in the two columns are very similar, suggesting a minimal effect of cross-province migration on our reference estimates.

**Table 7:** Models 3 estimates of scarring and trend effects. College graduates. Alternative specifications

	Real monthly earnings		
	Model 3	Province of 1st job	Region of birth
U0 x (*e* = 0)	0.005***	0.009***	0.001
	(0.002)	(0.002)	(0.003)
U0 x (*e* = 4)	−0.002	−0.001	−0.009***
	(0.001)	(0.002)	(0.002)
U0 x (*e* = 9)	0.001	− 0.001	− 0.006**
	(0.002)	(0.002)	(0.003)
Trend x (*e* = 0)	−0.016***	−0.016***	−0.025***
	(0.003)	(0.005)	(0.001)
Trend x (*e* = 4)	−0.005*	−0.004	−0.015***
	(0.003)	(0.005)	(0.001)
Trend x (*e* = 9)	−0.003	−0.006	−0.014***
	(0.003)	(0.005)	(0.002)
National UR	−0.007***	−0.008***	−0.007***
	(0.001)	(0.001)	(0.001)
Constant	6.498***	6.536***	6.483***
	(0.054)	(0.061)	(0.060)
Observations	9929	8887	4125
*R*^2^	0.812	0.840	0.887

	Days worked by worker		
	Model 3	Province of 1st job	Region of birth
U0 x (*e* = 0)	0.692***	1.179***	1.294***
	(0.238)	(0.311)	(0.379)
U0 x (*e* = 4)	−0.695***	−0.898***	−0.812**
	(0.188)	(0.28)	(0.319)
U0 x (*e* = 9)	0.245	0.036	0.615
	(0.209)	(0.380)	(0.390)
Trend x (*e* = 0)	−2.087***	−1.632***	−3.347***
	(0.790)	(0.566)	(0.192)
Trend x (*e* = 4)	−0.483	−0.462	−1.465***
	(0.79)	(0.551)	(0.161)
Trend x (*e* = 9)	−0.449	−0.949	−1.541***
	(0.819)	(0.663)	(0.264)
National UR	−1.181***	−0.294**	−1.150***
	(0.122)	(0.135)	(0.131)
Constant	218.16***	151.915***	216.551***
	(7.545)	(9.866)	(7.886)
Observations	9929	8887	4125
*R*^2^	0.806	0.805	0.890

The sample period is 1987–2019 except in the fourth column. The model includes fixed effects for the province of birth (province of first job in the second column and region of birth in the third column), years elapsed since graduation, and graduation cohort. National UR denotes the national unemployment rate. Standard errors clustered at the graduation year-province level are in parentheses. ***p < 0.01; **p < 0.05; *p < 0.10

**Fig. 8:**
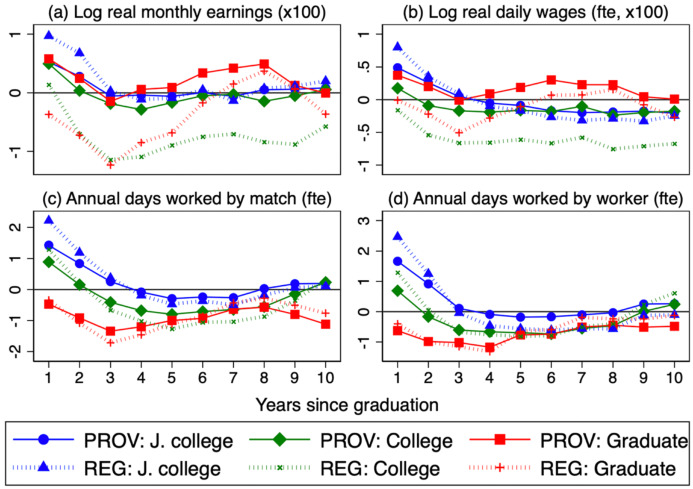
Estimated scarring effects in Model 3 with provincial and regional unemployment rates. *Notes*: The figure shows the scarring effects estimated for the three education groups in Model 3, namely the coefficients on the unemployment rate in the year of graduation in the province of birth (labeled “PROV” and the region of birth (“REG”), interacted with fixed effects for the years of potential experience, from the first (0) to the tenth (9). The symbols correspond to: (1) PROV Model: Junior college (blue circle), College (green diamond), and Graduate studies (red square). (2) REG Model: Junior college (blue triangle), College (green x sign), and Graduate studies (red plus sign) (colour figure online). The coefficients for monthly earnings and daily wages are multiplied by 100. All outcomes except for earnings are full time equivalents (fte)

The third column uses initial unemployment rates in the region of birth, as opposed to the province, to capture the scarring effects. For annual days worked, either by match or by worker, the estimates of scarring effects are virtually the same as in our reference model. On the other hand, for earnings the scarring effects are higher, more significant, and persistent. Figure 8 represents these effects with provincial and regional unemployment rates upon graduation. Focusing on college graduates and monthly earnings, with regional rates, the scarring effects peak in the fourth year, at − 1.1 pp, and they are highly significant until the tenth year, whereas with provincial rates, the effect in the fourth year is equal to − 0.3 pp and it is the only significant one, at the 10% level (apart from the first-year effect).^17^ These estimates are still well below those found by Fernández-Kranz and Rodríguez-Planas (2018) for Spain (see Sect. 4.4).

On the other hand, the estimated trend deterioration is also larger and more persistent if we consider the region as the relevant local labor market. For example, in the year of entry, the yearly fall in monthly earnings for college graduates is 2.5 pp vis-à-vis 1.6 pp with provincial unemployment rates. This means that in the first case, entering the labor market 10 years later now implies 25% lower monthly earnings vis-à-vis 16% in the model with provincial unemployment rates. The gap between the two estimates keeps on growing over time, with the regional specification stabilizing at a trend effect of 1.4 pp and the provincial specification stabilizing at 0.3 pp.

Naturally, the cumulative impact of the estimated effects is larger in this regional model than in the provincial one. We again add up the coefficients on the yearly estimates over the ten-year horizon, considering only the significant coefficients and discount future values. The 10-year impact of the scarring effects is now equal to 6.7% for each extra point increase in the regional unemployment rate, while it was nil in the provincial model.

Similarly, the ten-year impact of the downward trend more than doubles, reducing monthly earnings by 15.9%, whereas it was equal to 5.6% in the provincial unemployment model. In the case of annual days by worker, the cumulated scarring effects are slightly reduced to 2 days less for each point of increase in the regional unemployment rate (it was 2.7 days in the provincial model), while total effect of the trend over the 10-year horizon is much higher, at 16.6 days, vis-à-vis 3.4 days in the reference model.

The differences we have found in both the scarring and the trend effects depending on the definition of the unemployment rate used brings into question which geographical unit is the relevant definition of the local labor market in the Spanish case. We leave this question open for future work.

## Conclusions

In this paper, we have provided an overview of the youth labor market in Spain over the last four decades and analyzed the importance of the labor market conditions at entry for the subsequent labor market outcomes of its university graduates. We have highlighted a strong deterioration in employment rates, earnings, wages, and access to stable jobs over this period. Moreover, we have shown that young graduates have not taken advantage of the upgrading in the demand for skills, as their weight in high skill jobs did not increase.

In our empirical analysis, we have found some evidence of scarring effects from adverse labor market conditions at entry, but the effects are smaller and less persistent than in most other studies. The explanation for this surprising result is a trend deterioration in the early labor market outcomes of university graduates. The proximate mechanism for this worsening trend is that in the aftermath of deep recessions, the labor market outcomes of entrants do not return to their pre-recession levels. We find significant declining trends in employment and wages over time that affect young workers with university degrees both at the time of entry to the labor market and during the first ten years of their working careers. Our results indicate that rather than causing a temporary change in the earnings and employment profiles of certain unlucky cohorts of university graduates, deep recessions seem to provoke permanent changes in the youth labor market that negatively affect the outcomes of all future cohorts too.^18^

Our findings echo the recent warning by the International Labor Office about the continuing decline in young people’s engagement in the labor market and the falling returns to tertiary education (ILO 2020). This suggests that the Spanish experience may not be unique so that an exploration for other countries would be warranted—and that this phenomenon may be rooted in global rather than Spanish-specific causes.

In our view, future research should change its orientation, moving away from the scarring effects of recessions to an analysis of the forces that have contributed to the trend decline in the labor market outcomes of university graduates in Spain. The finding that the negative trends are larger in the first few years in the labor market suggests the presence of increasing problems in the school-to-work transition of Spanish youth. One explanation that is consistent with the evidence presented here is the endogenous nature of labor reforms. The strong deterioration in the labor market outcomes of youth led the Spanish authorities to implement reforms during recessions that introduced more flexible rules for the hiring and firing of young workers. However, other potential explanations are an increase in the degree of heterogeneity of the skill of university graduates due to the large increase in their numbers over time and skill-biased technological change that increases the demand for skills other than those provided by the educational system, which delivers a composition of the cohorts of university graduates by fields of study with a low weight of STEM disciplines. As always, more research is needed.

### Electronic supplementary material

Below is the link to the electronic supplementary material.

Supplementary file 1.

## Appendix 1: Variable description

Our analysis of employment spells, their duration, and the earnings of university graduates after their graduation is based on a combination of data from the Continuous Sample of Working Lives (CSWL) for 2019 and the Labor Force Survey (LFS) for the period 1987–2019.

*Date of graduation* The CSWL does not provide information on individuals’ date of graduation. Thus, we obtain information on the age of graduation from the LFS and impute it to each individual in the CSWL. Specifically, from the LFS microdata files for 2014Q3-2020Q3 (every 6 quarters, which is the maximum period per individual, so that nobody appears more than once), we calculate the median year of graduation for each cell defined by the educational level (junior college, college, and graduate degree), gender, and the province and year of birth. The reason is that the average duration of the degrees depends not only on its level, but also on the field of study, which in turn depends on the following variables: (1) The local offering of degrees: we assume that no mobility occurs until graduation, which is a sensible assumption since, according to the LFS, over the last 30 years almost 90% of all university students did their studies in their province of birth. (2) Gender: for example, there are more female students in health sciences and less in engineering. (3) Cohort: there have been changes in educational demand over time, but also changes in educational systems. In particular, due to the so-called Bologna process, college degree duration was reduced from five to four years and junior college duration was raised from three to four years. The generated database thus includes all years of degrees by educational level, year and province of birth and gender, from the year 1987 to the year 2019.

*Days worked and earnings* We construct a panel of employer-employee matches or self-employment spells for every month since the year of graduation (year 0). To create this panel, for each month, we compute the number of days worked in the match, the full-time equivalent days worked (i.e., days weighted by the coefficient of part-time work), the type of contract, the professional category, the monthly tax base (amount of earnings on which the social security tax is levied, which can be top and bottom coded), and, dividing by the number of days worked, the daily and full-time equivalent daily wages. The cells used in our analysis are obtained by collapsing this monthly information by year of graduation, calendar year, year of birth, province of birth, and gender, in the following way. (1) *Earnings:* in each cell, median wages are computed from the maximum (monthly or daily) tax base of each match in the 12 months of each calendar year. (2) *Days worked:* days of work in each match (only for employees) and days worked by each worker in all her/his jobs (including both as employee and self-employed) are computed as the average of the sum of all days worked in each match or for each individual in the calendar year. Earnings are deflated using the consumer price index with 1987 as the base year.

## Appendix 2: Decomposing the impact of cohort effects and national unemployment

See Table 8.

**Table 8:** Estimates of scarring and trend effects. College education. Decomposition of impact of the additional components of Model 3

	Log real monthly earnings			
	Model 1	Model 2	Model 3a	Model 3
U0 x (*e* = 0)	−0.010***	−0.007***	0.001	0.005***
	(0.002)	(0.001)	(0.002)	(0.002)
U0 x (*e* = 4)	−0.012***	−0.009***	−0.002	−0.002
	(0.001)	(0.001)	(0.001)	(0.001)
U0 x (*e* = 9)	0.001	−0.004***	0.004**	0.001
	(0.001)	(0.001)	(0.002)	(0.002)
Trend x (*e* = 0)		−0.0230***	−0.015***	−0.016***
		(0.001)	(0.003)	(0.003)
Trend x (*e* = 4)		−0.013***	−0.003	−0.005*
		(0.001)	(0.003)	(0.003)
Trend x (*e* = 9)		−0.013***	−0.002	−0.003
		(0.001)	(0.003)	(0.003)
National UR				−0.007***
				(0.001)
Constant	6.037***	6.444***	6.411***	6.498***
	(0.047)	(0.043)	(0.054)	(0.054)
Observations	9929	9929	9929	9929
*R*^2^	0.689	0.799	0.808	0.812

	Days worked by worker			
	Model 1	Model 2	Model 3a	Model 3
U0 x (*e* = 0)	−0.647***	− 0.288	0.061	0.692***
	(0.207)	(0.210)	(0.245)	(0.238)
U0 x (*e* = 4)	−1.240***	−1.150***	−0.746***	−0.695***
	(0.140)	(0.163)	(0.191)	(0.188)
U0 x (*e* = 9)	0.406***	0.107	0.739***	0.245
	(0.131)	(0.149)	(0.203)	(0.209)
Trend x (*e* = 0)		−2.332***	−1.830**	−2.087***
		(0.164)	(0.791)	(0.790)
Trend x (*e* = 4)		−0.534***	−0.134	−0.483
		(0.143)	(0.789)	(0.790)
Trend x (*e* = 9)		−0.768***	−0.260	−0.449
		(0.133)	(0.811)	(0.819)
National UR				−1.181***
				(0.122)
Constant	150.286***	191.510***	203.864***	218.160***
	(60.048)	(6.638)	(7.645)	(7.545)
Observations	9929	9929	9929	9929
*R*^2^	0.764	0.785	0.799	0.806

The sample period is 1987–2019. The model includes fixed effects for the province of birth and years elapsed since graduation. Standard errors clustered at the graduation year-province level are in parentheses. ****p* < 0.01; ***p* < 0.05; **p* < 0.10

## Footnotes

1 The list of countries includes Australia (Andrews et al. 2020), Austria (Brunner and Kuhn 2014), Belgium (Cockx and Girelli 2015), Germany (Arellano-Bover 2021), Japan (Genda, Kondo, and Ohta 2010) and Norway (Raaum and Roed 2006).

2 Some recent studies have looked at outcomes other than earnings or employment. Gorjón et al. (2021) analyze the persistence of initial precarious working conditions, while Arellano-Bover (2020) studies the long-term effects of landing a first job at a large firm versus a small one. Finally, Arellano-Bover (2020) show that income risk inequality between young and old individuals increases markedly during recessions.

3 For a comparison across European countries see Dolado (2015).

4 We informally use an extended definition of youth, going up to 29 years old, as opposed to the standard definition that stops at 24 years old.

5 For more information on international comparisons of unemployment fluctuations and some of their determining factors, see Hernanz and Jimeno (2017).

6 It is often argued that Spain is not an outlier if one considers relative rather than absolute differences between the youth and adult unemployment rates. However, the fact that the ratio between these rates is approximately constant and equal to the European average by no means implies that the high volatility in the youth unemployment rate is simply due to large movements in the adult unemployment rate. Indeed, it is unclear how one should rank countries based on the ratio between the youth and adult unemployment rate, as in several of the best-performing countries this ratio is substantially higher than in Spain.

7 Note that, due to this choice of sample, we are not reporting longitudinal variables, since individual workers move up across lines as they age, and the data are affected by worker attrition.

8 Detailed descriptions of these variables appear in the next section and in “Appendix 1”.

9 Note that the data for junior college stop in 2013, as this type of degree was abolished.

10 We also construct cohorts using the province of residence at first employment. As shown in next Section, nothing qualitatively important changes under this alternative definition.

11 We use the above definitions, as opposed to simple averages, because we think that they are more representative of the workers’ situation in a context in which most young workers have many short employment spells with the same or different firms within a year.

12 Our initial sample is a 4% random extraction from the population of Spanish workers. However, since we construct our sample backwards from the last year (2019), older cohorts are observed for longer lengths of time in the labor market, which imparts a systematic source of cell-size variation.

13 If scarring effects were originated at the national level, it would be impossible to estimate them separately from the impact of the national unemployment rate as a measure of business cycle conditions.

14 Notice that cohort fixed effects are not the same as time fixed effects, as the duration of university studies changes over time.

15 Also, the share of people aged 25–34 years old with university degrees who reside in their province of birth averages 80% for the period 1992–2020 according to the LFS.

16 As indicated, we are relying on the low mobility of Spanish young workers to define the province of birth as the one determining the relevant unemployment rate. To gauge the sensitivity of our results to this assumption, we also estimate the model with the province of residence at first employment instead. The estimates for this alternative specification, shown in Appendix 2 are practically identical to those reported in the text.

17 The full estimation results are presented in Table A5 of the Online Appendix.

18 In our empirical model we have assumed linear trends, so that they are independent of the scarring effects. In particular, we have not allowed them to depend directly on either the depth or the length of the recessions, whereas they are probably related in reality. This avenue is left for future work.
