# Supplementary material for: Lost in recessions: youth employment and earnings in Spain
Source: SERIEs (Berl). 2021 Nov 26;13(1-2):11–49. doi: 10.1007/s13209-021-00244-6 (PMC8625678; doi:10.1007/s13209-021-00244-6)
Supplement: Supplementary file 1 [file 13209_2021_244_MOESM1_ESM.docx]

**Lost in Recessions: Youth Employment and Earnings in Spain**

**Samuel Bentolila**

**CEMFI**

**Florentino Felgueroso**

**Fedea**

**Marcel Jansen**

**Universidad Autónoma de Madrid**

**Juan F. Jimeno**

**Banco de España and Universidad de Alcalá**

**28 July 2021**

**ONLINE APPENDIX**

**Table A1. Model 1 estimates of scarring effects**

|  | **Log real monthly earnings** | | | **Log real daily wages** | | | |
| --- | --- | --- | --- | --- | --- | --- | --- |
|  | Junior college | College | Graduate | Junior college | College | Graduate | |
| U0 x (e = 0) | .003 | -.01*** | -.025*** | .002 | -.004*** | -.014*** | |
|  | (.002) | (.002) | (.003) | (.001) | (.001) | (.002) | |
| U0 x (e = 1) | .001 | -.014*** | -.026*** | 0 | -.006*** | -.015*** | |
|  | (.002) | (.002) | (.003) | (.001) | (.001) | (.002) | |
| U0 x (e = 2) | -.002 | -.015*** | -.028*** | -.001 | -.006*** | -.016*** | |
|  | (.002) | (.001) | (.002) | (.001) | (.001) | (.001) | |
| U0 x (e = 3) | -.001 | -.015*** | -.025*** | -.001 | -.006*** | -.014*** | |
|  | (.001) | (.001) | (.002) | (.001) | (.001) | (.001) | |
| U0 x (e = 4) | -.001 | -.012*** | -.022*** | -.001 | -.005*** | -.012*** | |
|  | (.001) | (.001) | (.002) | (.001) | (.001) | (.001) | |
| U0 x (e = 5) | 0 | -.008*** | -.018*** | -.001 | -.004*** | -.01*** | |
|  | (.001) | (.001) | (.002) | (.001) | (.001) | (.001) | |
| U0 x (e = 6) | 0 | -.006*** | -.013*** | -.001 | -.002*** | -.008*** | |
|  | (.001) | (.001) | (.002) | (.001) | (.001) | (.001) | |
| U0 x (e = 7) | .002* | -.005*** | -.01*** | 0 | -.003*** | -.007*** | |
|  | (.001) | (.001) | (.002) | (.001) | (.001) | (.001) | |
| U0 x (e = 8) | .004*** | -.002 | -.009*** | .001 | -.001 | -.006*** | |
|  | (.001) | (.001) | (.002) | (.001) | (.001) | (.002) | |
| U0 x (e = 9) | .005*** | .001 | -.007*** | .001 | 0 | -.004*** | |
|  | (.001) | (.001) | (.002) | (.001) | (.001) | (.001) | |
| Constant | 5.902*** | 6.037*** | 6.786*** | 3.098*** | 3.183*** | 3.605*** | |
|  | (.05) | (.047) | (.087) | (.024) | (.02) | (.052) | |
| Observations | 8809 | 9929 | 7134 | 8808 | 9929 | 7132 | |
| R-squared | .535 | .689 | .457 | .52 | .716 | .444 | |
|  | **Annual days worked by match** | | | **Annual days worked by worker** | | | |
|  | Junior college | College | Graduate | Junior college | College | Graduate | |
| U0 x (e = 0) | 1.996*** | -.02 | -2.55*** | 1.251*** | -.647*** | -2.679*** | |
|  | (.288) | (.207) | (.314) | (.314) | (.207) | (.275) | |
| U0 x (e = 1) | 1.365*** | -.732*** | -2.996*** | .455* | -1.309*** | -2.762*** | |
|  | (.258) | (.187) | (.296) | (.274) | (.172) | (.229) | |
| U0 x (e = 2) | .642*** | -1.412*** | -3.391*** | -.387 | -1.571*** | -2.606*** | |
|  | (.246) | (.174) | (.288) | (.249) | (.154) | (.213) | |
| U0 x (e = 3) | .245 | -1.734*** | -3.501*** | -.501** | -1.417*** | -2.737*** | |
|  | (.243) | (.163) | (.297) | (.222) | (.154) | (.214) | |
| U0 x (e = 4) | -.124 | -1.824*** | -3.558*** | -.461** | -1.24*** | -2.316*** | |
|  | (.216) | (.163) | (.257) | (.195) | (.14) | (.208) | |
| U0 x (e = 5) | -.041 | -1.609*** | -3.427*** | -.214 | -1.02*** | -2.028*** | |
|  | (.197) | (.165) | (.267) | (.183) | (.125) | (.209) | |
| U0 x (e = 6) | -.147 | -1.38*** | -2.834*** | -.019 | -.653*** | -1.585*** | |
|  | (.204) | (.164) | (.297) | (.18) | (.116) | (.227) | |
| U0 x (e = 7) | .125 | -1.108*** | -2.46*** | .159 | -.387*** | -1.263*** | |
|  | (.207) | (.192) | (.303) | (.177) | (.137) | (.217) | |
| U0 x (e = 8) | .415** | -.541*** | -2.14*** | .648*** | .123 | -.885*** | |
|  | (.209) | (.197) | (.379) | (.177) | (.137) | (.282) | |
| U0 x (e = 9) | .572*** | -.1 | -1.819*** | .724*** | .406*** | -.373 | |
|  | (.193) | (.182) | (.442) | (.156) | (.131) | (.335) | |
| Constant | 71.138*** | 82.539*** | 205.413*** | 127.772*** | 150.286*** | 282.182*** | |
|  | (8.216) | (6.803) | (11.329) | (8.288) | (6.048) | (11.334) | |
| Observations | 8809 | 9929 | 7134 | 8809 | 9929 | 7134 | |
| R-squared | .524 | .662 | .377 | .636 | .764 | .399 | |
| Note. The sample period is 1987-2019 for college and graduate degrees. For junior college workers it is 1987-2013. The model includes fixed effects for the province of birth and years elapsed since graduation. Standard errors are in parentheses. *** *p*<.01, ** *p*<.05, * *p*<.10. | | | | | | |  |

**Table A2. Model 2 estimates of scarring and trend effects**

|  | **Log real monthly earnings** | | | **Log real daily wages** | | | |
| --- | --- | --- | --- | --- | --- | --- | --- |
|  | Junior college | College | Graduate | Junior college | College | Graduate | |
| U0 x (e = 0) | -.005** | -.007*** | -.01*** | .002 | -.003*** | -.006*** | |
|  | (.002) | (.001) | (.002) | (.001) | (.001) | (.001) | |
| U0 x (e = 1) | -.007*** | -.011*** | -.013*** | -.001 | -.005*** | -.007*** | |
|  | (.002) | (.001) | (.002) | (.001) | (.001) | (.001) | |
| U0 x (e = 2) | -.01*** | -.012*** | -.015*** | -.002** | -.005*** | -.008*** | |
|  | (.002) | (.001) | (.002) | (.001) | (.001) | (.001) | |
| U0 x (e = 3) | -.009*** | -.011*** | -.013*** | -.003*** | -.005*** | -.007*** | |
|  | (.001) | (.001) | (.002) | (.001) | (.001) | (.001) | |
| U0 x (e = 4) | -.008*** | -.009*** | -.012*** | -.002*** | -.004*** | -.006*** | |
|  | (.001) | (.001) | (.001) | (.001) | (.001) | (.001) | |
| U0 x (e = 5) | -.006*** | -.007*** | -.009*** | -.003*** | -.004*** | -.005*** | |
|  | (.001) | (.001) | (.001) | (.001) | (.001) | (.001) | |
| U0 x (e = 6) | -.005*** | -.006*** | -.007*** | -.002*** | -.003*** | -.005*** | |
|  | (.001) | (.001) | (.001) | (.001) | (.001) | (.001) | |
| U0 x (e = 7) | -.004*** | -.007*** | -.005*** | -.002* | -.004*** | -.004*** | |
|  | (.001) | (.001) | (.002) | (.001) | (.001) | (.001) | |
| U0 x (e = 8) | -.004*** | -.006*** | -.006*** | -.002* | -.003*** | -.004*** | |
|  | (.001) | (.001) | (.002) | (.001) | (.001) | (.001) | |
| U0 x (e = 9) | -.004*** | -.004*** | -.006*** | -.002** | -.003*** | -.003*** | |
|  | (.001) | (.001) | (.002) | (.001) | (.001) | (.001) | |
| Year trend x (e = 0) | -.024*** | -.023*** | -.048*** | .004*** | -.007*** | -.027*** | |
|  | (.002) | (.001) | (.002) | (.001) | (.001) | (.002) | |
| Year trend x (e = 1) | -.023*** | -.022*** | -.039*** | 0 | -.005*** | -.025*** | |
|  | (.002) | (.001) | (.002) | (.001) | (.001) | (.001) | |
| Year trend x (e = 2) | -.02*** | -.02*** | -.034*** | -.002** | -.005*** | -.021*** | |
|  | (.001) | (.001) | (.001) | (.001) | (.001) | (.001) | |
| Year trend x (e = 3) | -.019*** | -.017*** | -.028*** | -.004*** | -.006*** | -.017*** | |
|  | (.001) | (.001) | (.001) | (.001) | (.001) | (.001) | |
| Year trend x (e = 4) | -.017*** | -.013*** | -.022*** | -.004*** | -.006*** | -.013*** | |
|  | (.001) | (.001) | (.001) | (.001) | (.001) | (.001) | |
| Year trend x (e = 5) | -.016*** | -.012*** | -.019*** | -.004*** | -.006*** | -.011*** | |
|  | (.001) | (.001) | (.002) | (.001) | (.001) | (.001) | |
| Year trend x (e = 6) | -.015*** | -.011*** | -.015*** | -.004*** | -.006*** | -.01*** | |
|  | (.001) | (.001) | (.001) | (.001) | (.001) | (.001) | |
| Year trend x (e = 7) | -.013*** | -.011*** | -.014*** | -.004*** | -.006*** | -.007*** | |
|  | (.001) | (.001) | (.001) | (.001) | (.001) | (.001) | |
| Year trend x (e = 8) | -.014*** | -.013*** | -.014*** | -.006*** | -.007*** | -.006*** | |
|  | (.001) | (.001) | (.001) | (.001) | (.001) | (.001) | |
| Year trend x (e = 9) | -.016*** | -.013*** | -.012*** | -.007*** | -.008*** | -.005*** | |
|  | (.001) | (.001) | (.001) | (.001) | (.001) | (.001) | |
| Constant | 6.361*** | 6.444*** | 7.725*** | 3.037*** | 3.301*** | 4.143*** | |
|  | (.061) | (.043) | (.078) | (.034) | (.023) | (.047) | |
| Observations | 8809 | 9929 | 7134 | 8808 | 9929 | 7132 | |
| R-squared | .641 | .799 | .655 | .538 | .755 | .625 | |
|  | | | | | | |  |

|  |
| --- |

**Table A2. Model 2 estimates of scarring and trend effects (continued)**

|  | **Annual days worked by match** | | | **Annual days worked by worker** | | |
| --- | --- | --- | --- | --- | --- | --- |
|  | Junior college | College | Graduate | Junior college | College | Graduate |
| U0 x (e = 0) | .628** | .416** | -.94*** | .223 | -.288 | -1.393*** |
|  | (.267) | (.185) | (.238) | (.307) | (.21) | (.237) |
| U0 x (e = 1) | .04 | -.346* | -1.423*** | -.475* | -1.06*** | -1.7*** |
|  | (.24) | (.179) | (.224) | (.262) | (.19) | (.208) |
| U0 x (e = 2) | -.558** | -.973*** | -1.92*** | -1.201*** | -1.378*** | -1.685*** |
|  | (.232) | (.178) | (.237) | (.24) | (.178) | (.215) |
| U0 x (e = 3) | -.917*** | -1.309*** | -1.975*** | -1.224*** | -1.275*** | -1.764*** |
|  | (.229) | (.182) | (.259) | (.216) | (.182) | (.209) |
| U0 x (e = 4) | -1.143*** | -1.496*** | -1.947*** | -1.089*** | -1.15*** | -1.291*** |
|  | (.208) | (.184) | (.213) | (.188) | (.163) | (.199) |
| U0 x (e = 5) | -1.113*** | -1.436*** | -1.987*** | -.877*** | -.992*** | -1.175*** |
|  | (.182) | (.169) | (.222) | (.172) | (.138) | (.205) |
| U0 x (e = 6) | -1.15*** | -1.415*** | -1.679*** | -.629*** | -.663*** | -.808*** |
|  | (.173) | (.143) | (.251) | (.166) | (.121) | (.21) |
| U0 x (e = 7) | -.918*** | -1.411*** | -1.595*** | -.451*** | -.49*** | -.683*** |
|  | (.176) | (.162) | (.256) | (.168) | (.141) | (.194) |
| U0 x (e = 8) | -.879*** | -1.154*** | -1.73*** | -.132 | -.081 | -.614** |
|  | (.198) | (.184) | (.311) | (.181) | (.146) | (.242) |
| U0 x (e = 9) | -.96*** | -.841*** | -1.865*** | -.171 | .107 | -.417 |
|  | (.195) | (.183) | (.322) | (.18) | (.149) | (.272) |
| Year trend x (e = 0) | -4.196*** | -3.044*** | -4.924*** | -3.464*** | -2.332*** | -4.135*** |
|  | (.372) | (.157) | (.29) | (.364) | (.164) | (.25) |
| Year trend x (e = 1) | -3.812*** | -2.631*** | -4.677*** | -2.859*** | -1.583*** | -3.193*** |
|  | (.3) | (.143) | (.232) | (.269) | (.15) | (.206) |
| Year trend x (e = 2) | -3.232*** | -2.432*** | -3.94*** | -2.316*** | -1.027*** | -2.438*** |
|  | (.273) | (.157) | (.239) | (.241) | (.145) | (.235) |
| Year trend x (e = 3) | -3.057*** | -2.251*** | -3.666*** | -1.895*** | -.737*** | -2.323*** |
|  | (.263) | (.167) | (.247) | (.209) | (.159) | (.225) |
| Year trend x (e = 4) | -2.557*** | -1.939*** | -3.863*** | -1.549*** | -.534*** | -2.446*** |
|  | (.213) | (.177) | (.253) | (.182) | (.143) | (.219) |
| Year trend x (e = 5) | -2.804*** | -1.828*** | -3.57*** | -1.711*** | -.344*** | -2.058*** |
|  | (.194) | (.178) | (.284) | (.172) | (.131) | (.239) |
| Year trend x (e = 6) | -2.588*** | -1.692*** | -3.32*** | -1.517*** | -.401*** | -2.257*** |
|  | (.177) | (.153) | (.29) | (.157) | (.111) | (.213) |
| Year trend x (e = 7) | -2.399*** | -1.873*** | -2.896*** | -1.296*** | -.663*** | -1.977*** |
|  | (.193) | (.167) | (.286) | (.154) | (.13) | (.234) |
| Year trend x (e = 8) | -2.441*** | -1.863*** | -3.113*** | -1.432*** | -.632*** | -2.266*** |
|  | (.196) | (.182) | (.332) | (.169) | (.124) | (.271) |
| Year trend x (e = 9) | -2.7*** | -1.861*** | -3.118*** | -1.515*** | -.768*** | -2.139*** |
|  | (.223) | (.184) | (.335) | (.182) | (.133) | (.29) |
| Constant | 152.736*** | 136.856*** | 303.717*** | 194.125*** | 191.509*** | 364.1*** |
|  | (11.699) | (6.908) | (10.41) | (11.236) | (6.638) | (10.799) |
| Observations | 8809 | 9929 | 7134 | 8809 | 9929 | 7134 |
| R-squared | .634 | .755 | .545 | .682 | .785 | .509 |
| Note. The sample period is 1987-2019 for college and graduate degrees. For junior college workers it is 1987-2013. The model includes fixed effects for the province of birth and years elapsed since graduation. Standard errors are in parentheses. *** *p*<.01, ** *p*<.05, * *p*<.10. | | | | | | |

**Table A3. Model 3 estimates of scarring and trend effects**

|  | **Log real monthly earnings** | | | **Log real daily wages** | | | |
| --- | --- | --- | --- | --- | --- | --- | --- |
|  | Junior college | College | Graduate | Junior college | College | Graduate | |
| U0 x (e = 0) | .005** | .005*** | .006* | .005*** | .002 | .004* | |
|  | (.002) | (.002) | (.003) | (.002) | (.001) | (.002) | |
| U0 x (e = 1) | .003 | 0 | .002 | .003 | -.001 | .002 | |
|  | (.002) | (.002) | (.003) | (.002) | (.001) | (.002) | |
| U0 x (e = 2) | -.001 | -.002 | -.001 | 0 | -.002 | 0 | |
|  | (.002) | (.002) | (.003) | (.002) | (.001) | (.002) | |
| U0 x (e = 3) | 0 | -.003* | .001 | -.001 | -.002* | .001 | |
|  | (.002) | (.002) | (.003) | (.002) | (.001) | (.002) | |
| U0 x (e = 4) | -.001 | -.002 | .001 | -.001 | -.002 | .002 | |
|  | (.002) | (.001) | (.003) | (.002) | (.001) | (.002) | |
| U0 x (e = 5) | 0 | 0 | .003 | -.002 | -.002* | .003 | |
|  | (.002) | (.001) | (.003) | (.001) | (.001) | (.002) | |
| U0 x (e = 6) | -.001 | 0 | .004 | -.002 | -.001 | .002 | |
|  | (.002) | (.002) | (.003) | (.001) | (.001) | (.002) | |
| U0 x (e = 7) | .001 | -.001 | .005 | -.002 | -.002** | .002 | |
|  | (.002) | (.002) | (.003) | (.001) | (.001) | (.002) | |
| U0 x (e = 8) | .001 | -.001 | .001 | -.002 | -.002* | 0 | |
|  | (.002) | (.002) | (.003) | (.002) | (.001) | (.002) | |
| U0 x (e = 9) | .001 | .001 | 0 | -.002 | -.002 | 0 | |
|  | (.002) | (.002) | (.003) | (.001) | (.001) | (.002) | |
| Year trend x (e = 0) | -.027*** | -.016*** | -.035*** | .002 | -.005*** | -.019*** | |
|  | (.002) | (.003) | (.003) | (.002) | (.002) | (.002) | |
| Year trend x (e = 1) | -.026*** | -.016*** | -.026*** | -.002 | -.003** | -.017*** | |
|  | (.002) | (.003) | (.003) | (.002) | (.002) | (.002) | |
| Year trend x (e = 2) | -.022*** | -.012*** | -.02*** | -.003* | -.003* | -.012*** | |
|  | (.002) | (.003) | (.003) | (.002) | (.002) | (.002) | |
| Year trend x (e = 3) | -.021*** | -.008*** | -.016*** | -.005*** | -.003* | -.009*** | |
|  | (.002) | (.003) | (.003) | (.002) | (.002) | (.002) | |
| Year trend x (e = 4) | -.019*** | -.005* | -.012*** | -.005*** | -.003* | -.007*** | |
|  | (.002) | (.003) | (.003) | (.002) | (.002) | (.002) | |
| Year trend x (e = 5) | -.018*** | -.004 | -.011*** | -.005*** | -.003 | -.006*** | |
|  | (.002) | (.003) | (.003) | (.001) | (.002) | (.002) | |
| Year trend x (e = 6) | -.017*** | -.003 | -.01*** | -.005*** | -.002 | -.006** | |
|  | (.002) | (.003) | (.003) | (.001) | (.002) | (.002) | |
| Year trend x (e = 7) | -.015*** | -.003 | -.011*** | -.005*** | -.002 | -.005** | |
|  | (.002) | (.003) | (.003) | (.002) | (.002) | (.002) | |
| Year trend x (e = 8) | -.015*** | -.003 | -.014*** | -.006*** | -.003* | -.006** | |
|  | (.002) | (.003) | (.003) | (.002) | (.002) | (.002) | |
| Year trend x (e = 9) | -.015*** | -.003 | -.013*** | -.007*** | -.004** | -.006** | |
|  | (.002) | (.003) | (.004) | (.002) | (.002) | (.002) | |
| National UR | -.008*** | -.007*** | -.007*** | -.005*** | -.004*** | -.004*** | |
|  | (.001) | (.001) | (.001) | (.001) | (.001) | (.001) | |
| Constant | 6.484*** | 6.498*** | 7.342*** | 3.148*** | 3.357*** | 3.907*** | |
|  | (.067) | (.054) | (.107) | (.042) | (.033) | (.07) | |
| Observations | 8809 | 9929 | 7134 | 8808 | 9929 | 7132 | |
| R-squared | .665 | .812 | .687 | .561 | .766 | .656 | |
|  | | | | | | |  |

**Table A3. Model 3 estimates of scarring and trend effects (continued)**

|  | **Annual days worked by match** | | | **Annual days worked by worker** | | | |
| --- | --- | --- | --- | --- | --- | --- | --- |
|  | Junior college | College | Graduate | Junior college | College | Graduate | |
| U0 x (e = 0) | 1.425*** | .891*** | -.47 | 1.665*** | .692*** | -.627 | |
|  | (.348) | (.233) | (.42) | (.366) | (.238) | (.397) | |
| U0 x (e = 1) | .838*** | .158 | -.915** | .917*** | -.165 | -.984** | |
|  | (.317) | (.22) | (.407) | (.328) | (.223) | (.388) | |
| U0 x (e = 2) | .258 | -.41* | -1.343*** | .108 | -.605*** | -1.02*** | |
|  | (.294) | (.211) | (.399) | (.297) | (.202) | (.384) | |
| U0 x (e = 3) | -.08 | -.679*** | -1.199*** | -.09 | -.663*** | -1.171*** | |
|  | (.284) | (.212) | (.405) | (.284) | (.203) | (.381) | |
| U0 x (e = 4) | -.292 | -.798*** | -.997** | -.179 | -.695*** | -.768** | |
|  | (.278) | (.209) | (.401) | (.279) | (.188) | (.374) | |
| U0 x (e = 5) | -.24 | -.709*** | -.928** | -.167 | -.733*** | -.74* | |
|  | (.262) | (.209) | (.416) | (.266) | (.186) | (.378) | |
| U0 x (e = 6) | -.263 | -.646*** | -.634 | -.103 | -.551*** | -.51 | |
|  | (.265) | (.212) | (.41) | (.267) | (.183) | (.382) | |
| U0 x (e = 7) | .03 | -.556** | -.568 | -.033 | -.462** | -.445 | |
|  | (.269) | (.224) | (.448) | (.267) | (.191) | (.392) | |
| U0 x (e = 8) | .188 | -.142 | -.801* | .253 | .012 | -.511 | |
|  | (.28) | (.245) | (.477) | (.275) | (.202) | (.427) | |
| U0 x (e = 9) | .2 | .235 | -1.118** | .263 | .245 | -.482 | |
|  | (.283) | (.241) | (.468) | (.284) | (.209) | (.464) | |
| Year trend x (e = 0) | -4.52*** | -3.032*** | -4.391*** | -4.174*** | -2.087*** | -3.945*** | |
|  | (.431) | (.422) | (.417) | (.431) | (.79) | (.519) | |
| Year trend x (e = 1) | -4.127*** | -2.569*** | -4.061*** | -3.367*** | -1.317* | -3.004*** | |
|  | (.397) | (.418) | (.404) | (.396) | (.789) | (.52) | |
| Year trend x (e = 2) | -3.576*** | -2.406*** | -3.338*** | -2.695*** | -.855 | -2.371*** | |
|  | (.39) | (.418) | (.438) | (.377) | (.789) | (.54) | |
| Year trend x (e = 3) | -3.416*** | -2.272*** | -3.335*** | -2.22*** | -.633 | -2.382*** | |
|  | (.385) | (.42) | (.427) | (.371) | (.789) | (.529) | |
| Year trend x (e = 4) | -2.94*** | -2.018*** | -3.831*** | -1.845*** | -.483 | -2.669*** | |
|  | (.372) | (.421) | (.427) | (.368) | (.79) | (.526) | |
| Year trend x (e = 5) | -3.183*** | -2.057*** | -3.853*** | -1.994*** | -.472 | -2.46*** | |
|  | (.374) | (.431) | (.455) | (.369) | (.796) | (.528) | |
| Year trend x (e = 6) | -3.003*** | -1.992*** | -4.026*** | -1.853*** | -.528 | -2.851*** | |
|  | (.372) | (.442) | (.456) | (.372) | (.797) | (.517) | |
| Year trend x (e = 7) | -2.742*** | -2.146*** | -3.802*** | -1.551*** | -.717 | -2.558*** | |
|  | (.42) | (.451) | (.479) | (.397) | (.8) | (.552) | |
| Year trend x (e = 8) | -2.647*** | -2.014*** | -4.206*** | -1.543*** | -.43 | -3.005*** | |
|  | (.424) | (.471) | (.509) | (.395) | (.806) | (.556) | |
| Year trend x (e = 9) | -2.777*** | -1.958*** | -4.369*** | -1.45*** | -.449 | -3.096*** | |
|  | (.443) | (.485) | (.488) | (.432) | (.819) | (.589) | |
| National UR | .101 | .387*** | .552*** | -1.518*** | -1.181*** | -.742*** | |
|  | (.155) | (.126) | (.165) | (.144) | (.122) | (.143) | |
| Constant | 161.716*** | 146.404*** | 269.724*** | 224.513*** | 218.161*** | 356.839*** | |
|  | (12.145) | (8.191) | (13.668) | (11.999) | (7.545) | (14.615) | |
| Observations | 8809 | 9929 | 7134 | 8809 | 9929 | 7134 | |
| R-squared | .659 | .777 | .576 | .705 | .806 | .525 | |
| \| Note. The sample period is 1987-2019 for college and graduate degrees. For junior college workers it is 1987-2013. The model includes fixed effects for the province of birth, years elapsed since graduation, and graduation cohort. Standard errors are in parentheses. *** *p*<.01, ** *p*<.05, * *p*<.10. \| \| --- \| | | | | | | |  |

**Table A4. Models 1, 2, and 3 estimates of scarring and trend effects by gender**

1. **Log of real monthly earnings**

|  | Junior college | College | Graduate | Junior college | College | Graduate |
| --- | --- | --- | --- | --- | --- | --- |
|  | Men | Men | Men | Women | Women | Women |
| \| **Model 1. Interactions of initial unemployment with experience** \| \| --- \| | | | | | | |
| U0 x (e = 0) | .002 | -.004*** | -.008*** | .001 | -.005*** | -.01*** |
|  | (.001) | (.001) | (.001) | (.001) | (.001) | (.001) |
| U0 x (e = 4) | .001 | -.003*** | -.006*** | -.002*** | -.005*** | -.008*** |
|  | (.001) | (.001) | (.001) | (.001) | (.001) | (.001) |
| U0 x (e = 9) | .002** | .001* | -.001 | .001* | .001 | -.001 |
|  | (.001) | (.001) | (.001) | (.001) | (.001) | (.001) |
| Constant | 6.152*** | 6.214*** | 6.757*** | 5.899*** | 5.92*** | 6.67*** |
|  | (.072) | (.049) | (.096) | (.061) | (.074) | (.088) |
| Observations | 7850 | 9458 | 6558 | 8674 | 9596 | 6994 |
| R-squared | .433 | .574 | .322 | .426 | .616 | .391 |
| **Model 2. Interactions of initial unemployment with experience and trends** | | | | | | |
| U0 x (e = 0) | 0 | -.003*** | -.003** | 0 | -.002** | -.003*** |
|  | (.001) | (.001) | (.001) | (.001) | (.001) | (.001) |
| U0 x (e = 4) | -.001 | -.002*** | -.003*** | -.004*** | -.004*** | -.003*** |
|  | (.001) | (.001) | (.001) | (.001) | (.001) | (.001) |
| U0 x (e = 9) | -.001 | 0 | -.001 | -.001 | 0 | 0 |
|  | (.001) | (.001) | (.001) | (.001) | (.001) | (.001) |
| Year trend x (e = 0) | -.018*** | -.024*** | -.035*** | -.021*** | -.022*** | -.042*** |
|  | (.002) | (.001) | (.003) | (.002) | (.002) | (.002) |
| Year trend x (e = 4) | -.015*** | -.014*** | -.017*** | -.014*** | -.012*** | -.019*** |
|  | (.002) | (.001) | (.002) | (.001) | (.001) | (.002) |
| Year trend x (e = 9) | -.013*** | -.011*** | -.006*** | -.012*** | -.008*** | -.009*** |
|  | (.001) | (.001) | (.002) | (.001) | (.001) | (.002) |
| Constant | 6.485*** | 6.666*** | 7.46*** | 6.257*** | 6.31*** | 7.477*** |
|  | (.08) | (.054) | (.106) | (.077) | (.067) | (.09) |
| Observations | 7850 | 9458 | 6558 | 8674 | 9596 | 6994 |
| R-squared | .493 | .672 | .475 | .503 | .699 | .535 |
| \| **Model 3. Interactions of initial unemployment with experience and trends, cohort fixed effects, national unemployment** \| \| --- \| | | | | | | |
| U0 x (e = 0) | .002 | .001 | .001 | .003*** | .002** | .001 |
|  | (.001) | (.001) | (.001) | (.001) | (.001) | (.001) |
| U0 x (e = 4) | 0 | .001 | 0 | -.001* | -.001** | 0 |
|  | (.001) | (.001) | (.001) | (.001) | (.001) | (.001) |
| U0 x (e = 9) | -.001 | 0 | -.001 | 0 | .002*** | .001 |
|  | (.001) | (.001) | (.001) | (.001) | (.001) | (.001) |
| Year trend x (e = 0) | -.02*** | -.019*** | -.027*** | -.026*** | -.011*** | -.035*** |
|  | (.003) | (.002) | (.003) | (.003) | (.003) | (.003) |
| Year trend x (e = 4) | -.015*** | -.008*** | -.011*** | -.018*** | .001 | -.014*** |
|  | (.003) | (.002) | (.003) | (.003) | (.003) | (.003) |
| Year trend x (e = 9) | -.011*** | -.008*** | -.01*** | -.013*** | .006* | -.011*** |
|  | (.003) | (.002) | (.003) | (.003) | (.003) | (.004) |
| National UR | -.009*** | -.006*** | -.006*** | -.007*** | -.005*** | -.008*** |
|  | (.001) | (.001) | (.001) | (.001) | (.001) | (.001) |
| Constant | 6.631*** | 6.714*** | 7.242*** | 6.486*** | 6.432*** | 7.387*** |
|  | (.086) | (.064) | (.1) | (.082) | (.069) | (.1) |
| Observations | 7850 | 9458 | 6558 | 8674 | 9596 | 6994 |
| R-squared | .515 | .693 | .522 | .534 | .72 | .571 |

**Table A4. Models 1, 2, and 3 estimates of scarring and trend effects by gender (continued)**

1. **Log of real full-time equivalent daily wage**

|  | | Junior college | | College | | Graduate | | Junior college | | College | | Graduate | |
| --- | --- | --- | --- | --- | --- | --- | --- | --- | --- | --- | --- | --- | --- |
|  | | Men | | Men | | Men | | Women | | Women | | Women | |
| \| **Model 1. Interactions of initial unemployment with experience** \| \| --- \| | | | | | | | | | | | | | |
| U0 x (e = 0) | | .001 | | -.002*** | | -.005*** | | .001 | | -.002*** | | -.006*** | |
|  | | (.001) | | (.001) | | (.001) | | (.001) | | (.001) | | (.001) | |
| U0 x (e = 4) | | 0 | | -.002*** | | -.004*** | | -.001** | | -.002*** | | -.004*** | |
|  | | (.001) | | (.001) | | (.001) | | (.001) | | (.001) | | (.001) | |
| U0 x (e = 9) | | .001 | | .001*** | | -.001 | | 0 | | 0 | | -.001 | |
|  | | (.001) | | (.001) | | (.001) | | (.001) | | (.001) | | (.001) | |
| Constant | | 3.204*** | | 3.218*** | | 3.594*** | | 3.137*** | | 3.176*** | | 3.556*** | |
|  | | (.032) | | (.029) | | (.049) | | (.044) | | (.043) | | (.056) | |
| Observations | | 7850 | | 9457 | | 6556 | | 8674 | | 9595 | | 6992 | |
| R-squared | | .419 | | .587 | | .296 | | .357 | | .605 | | .353 | |
| **Model 2. Interactions of initial unemployment with experience and trends** | | | | | | | | | | | | | |
| U0 x (e = 0) | | .001 | | -.002*** | | -.002** | | .001* | | -.001*** | | -.002*** | |
|  | | (.001) | | (.001) | | (.001) | | (.001) | | (.001) | | (.001) | |
| U0 x (e = 4) | | -.001 | | -.001*** | | -.002*** | | -.001** | | -.002*** | | -.002*** | |
|  | | (.001) | | (.001) | | (.001) | | (.001) | | (.001) | | (.001) | |
| U0 x (e = 9) | | -.001 | | 0 | | 0 | | -.001 | | 0 | | -.001 | |
|  | | (.001) | | (.001) | | (.001) | | (.001) | | (.001) | | (.001) | |
| Year trend x (e = 0) | | .004*** | | -.01*** | | -.021*** | | .005*** | | -.004*** | | -.021*** | |
|  | | (.001) | | (.001) | | (.002) | | (.001) | | (.001) | | (.001) | |
| Year trend x (e = 4) | | -.007*** | | -.008*** | | -.011*** | | -.001 | | -.003*** | | -.01*** | |
|  | | (.001) | | (.001) | | (.001) | | (.001) | | (.001) | | (.001) | |
| Year trend x (e = 9) | | -.009*** | | -.006*** | | -.002* | | -.002** | | -.003*** | | -.007*** | |
|  | | (.001) | | (.001) | | (.001) | | (.001) | | (.001) | | (.001) | |
| Constant | | 3.148*** | | 3.402*** | | 4.02*** | | 3.059*** | | 3.243*** | | 3.955*** | |
|  | | (.039) | | (.035) | | (.058) | | (.045) | | (.045) | | (.061) | |
| Observations | | 7850 | | 9457 | | 6556 | | 8674 | | 9595 | | 6992 | |
| R-squared | | .444 | | .639 | | .429 | | .359 | | .618 | | .457 | |
| **Model 3. Interactions of initial unemployment with experience and trends, cohort fixed effects, national unemployment** | | | | | | | | | | | | | |
| U0 x (e = 0) | | .002** | | 0 | | .001 | | .002*** | | 0 | | 0 | |
|  | | (.001) | | (.001) | | (.001) | | (.001) | | (.001) | | (.001) | |
| U0 x (e = 4) | | -.001 | | 0 | | .001 | | 0 | | -.001* | | -.001 | |
|  | | (.001) | | (.001) | | (.001) | | (.001) | | (.001) | | (.001) | |
| U0 x (e = 9) | | -.001** | | 0 | | 0 | | 0 | | .001 | | 0 | |
|  | | (.001) | | (.001) | | (.001) | | (.001) | | (.001) | | (.001) | |
| Year trend x (e = 0) | | .004* | | -.009*** | | -.016*** | | .003 | | -.003** | | -.017*** | |
|  | | (.002) | | (.001) | | (.002) | | (.002) | | (.001) | | (.002) | |
| Year trend x (e = 4) | | -.005*** | | -.008*** | | -.008*** | | -.002 | | -.001 | | -.007*** | |
|  | | (.002) | | (.002) | | (.002) | | (.002) | | (.001) | | (.002) | |
| Year trend x (e = 9) | | -.006*** | | -.007*** | | -.006** | | -.002 | | 0 | | -.007*** | |
|  | | (.002) | | (.002) | | (.002) | | (.002) | | (.001) | | (.002) | |
| National UR | | -.006*** | | -.004*** | | -.005*** | | -.002*** | | -.002*** | | -.003*** | |
|  | | (.001) | | (.001) | | (.001) | | (.001) | | (.001) | | (.001) | |
| Constant | | 3.238*** | | 3.448*** | | 3.891*** | | 3.181*** | | 3.318*** | | 3.893*** | |
|  | | (.054) | | (.045) | | (.061) | | (.05) | | (.048) | | (.073) | |
| Observations | | 7850 | | 9457 | | 6556 | | 8674 | | 9595 | | 6992 | |
| R-squared | | .464 | | .656 | | .471 | | .396 | | .634 | | .491 | |

**Table A4. Models 1, 2, and 3 estimates of scarring and trend effects by gender (continued)**

1. **Annual full-time equivalent days worked by match**

|  | | Junior college | | College | | Graduate | | Junior college | | College | | Graduate | |
| --- | --- | --- | --- | --- | --- | --- | --- | --- | --- | --- | --- | --- | --- |
|  | | Men | | Men | | Men | | Women | | Women | | Women | |
| \| \| **Model 1. Interactions of initial unemployment with experience** \| \| --- \| \| \| --- \| --- \| | | | | | | | | | | | | | |
| U0 x (e = 0) | | .946*** | | -.144 | | -.876*** | | .633*** | | -.089 | | -1.151*** | |
|  | | (.194) | | (.125) | | (.166) | | (.146) | | (.121) | | (.161) | |
| U0 x (e = 4) | | -.049 | | -.7*** | | -1.186*** | | -.26** | | -.876*** | | -1.362*** | |
|  | | (.136) | | (.107) | | (.143) | | (.112) | | (.091) | | (.145) | |
| U0 x (e = 9) | | -.038 | | .027 | | -.27 | | .009 | | .079 | | -.427* | |
|  | | (.121) | | (.108) | | (.215) | | (.114) | | (.108) | | (.221) | |
| Constant | | 124.136*** | | 120.545*** | | 221.612*** | | 80.47*** | | 79.509*** | | 206.541*** | |
|  | | (15.998) | | (6.839) | | (12.609) | | (9.359) | | (8.984) | | (11.927) | |
| Observations | | 7850 | | 9459 | | 6558 | | 8674 | | 9596 | | 6994 | |
| R-squared | | .376 | | .513 | | .224 | | .441 | | .583 | | .279 | |
| **Model 2. Interactions of initial unemployment with experience and trends** | | | | | | | | | | | | | |
| U0 x (e = 0) | | .619*** | | -.022 | | -.252 | | .343*** | | .256** | | -.269* | |
|  | | (.187) | | (.103) | | (.158) | | (.117) | | (.113) | | (.144) | |
| U0 x (e = 4) | | -.236* | | -.558*** | | -.522*** | | -.492*** | | -.684*** | | -.477*** | |
|  | | (.132) | | (.096) | | (.137) | | (.107) | | (.102) | | (.136) | |
| U0 x (e = 9) | | -.364*** | | -.13 | | -.294 | | -.44*** | | -.155 | | -.351* | |
|  | | (.126) | | (.114) | | (.183) | | (.109) | | (.109) | | (.189) | |
| Year trend x (e = 0) | | -3.406*** | | -2.669*** | | -3.964*** | | -3.722*** | | -3.234*** | | -4.79*** | |
|  | | (.393) | | (.194) | | (.349) | | (.307) | | (.194) | | (.314) | |
| Year trend x (e = 4) | | -1.79*** | | -1.764*** | | -3.381*** | | -2.691*** | | -1.909*** | | -4.004*** | |
|  | | (.332) | | (.193) | | (.33) | | (.253) | | (.201) | | (.303) | |
| Year trend x (e = 9) | | -1.825*** | | -1.077*** | | -2.305*** | | -2.652*** | | -1.53*** | | -2.951*** | |
|  | | (.285) | | (.242) | | (.371) | | (.252) | | (.226) | | (.371) | |
| Constant | | 185.982*** | | 170.291*** | | 304.403*** | | 145.001*** | | 136.541*** | | 300.237*** | |
|  | | (16.506) | | (8.132) | | (14.636) | | (12.968) | | (8.511) | | (11.852) | |
| Observations | | 7850 | | 9459 | | 6558 | | 8674 | | 9596 | | 6994 | |
| R-squared | | .421 | | .578 | | .353 | | .541 | | .67 | | .429 | |
| \| **Model 3. Interactions of initial unemployment with experience and trends, cohort fixed effects, national**  **unemployment** \| \| --- \| | | | | | | | | | | | | | |
| U0 x (e = 0) | | .79*** | | .062 | | -.247 | | .489*** | | .506*** | | -.065 | |
|  | | (.202) | | (.123) | | (.188) | | (.128) | | (.117) | | (.182) | |
| U0 x (e = 4) | | -.072 | | -.3*** | | -.239 | | -.311*** | | -.314*** | | -.12 | |
|  | | (.137) | | (.107) | | (.176) | | (.109) | | (.095) | | (.168) | |
| U0 x (e = 9) | | -.063 | | .253* | | -.028 | | -.126 | | .386*** | | .011 | |
|  | | (.148) | | (.131) | | (.24) | | (.123) | | (.125) | | (.217) | |
| Year trend x (e = 0) | | -3.846*** | | -2.861*** | | -3.195*** | | -4.014*** | | -2.781*** | | -3.23*** | |
|  | | (.534) | | (.339) | | (.342) | | (.458) | | (.406) | | (.447) | |
| Year trend x (e = 4) | | -2.366*** | | -2.025*** | | -2.974*** | | -3.065*** | | -1.509*** | | -2.666*** | |
|  | | (.471) | | (.352) | | (.387) | | (.442) | | (.412) | | (.478) | |
| Year trend x (e = 9) | | -2.115*** | | -1.916*** | | -3.213*** | | -2.708*** | | -1.375*** | | -2.708*** | |
|  | | (.553) | | (.443) | | (.509) | | (.479) | | (.472) | | (.513) | |
| National UR | | .097 | | .735*** | | .922*** | | .121 | | .525*** | | .464** | |
|  | | (.181) | | (.154) | | (.202) | | (.151) | | (.136) | | (.204) | |
| Constant | | 195.78*** | | 172.558*** | | 247.919*** | | 165.497*** | | 145.19*** | | 247.833*** | |
|  | | (17.01) | | (9.561) | | (14.646) | | (12.886) | | (9.942) | | (13.474) | |
| Observations | | 7850 | | 9459 | | 6558 | | 8674 | | 9596 | | 6994 | |
| R-squared | | .447 | | .608 | | .396 | | .57 | | .692 | | .452 | |

**Table A4. Models 1, 2, and 3 estimates of scarring and trend effects by gender (continued)**

1. **Annual full-time equivalent days worked by worker**

|  | Junior college | College | Graduate | Junior college | College | Graduate |
| --- | --- | --- | --- | --- | --- | --- |
|  | Men | Men | Men | Women | Women | Women |
| \| **Model 1. Interactions of initial unemployment with experience** \| \| --- \| | | | | | | |
| U0 x (e = 0) | .619*** | -.333*** | -1.107*** | .386** | -.379*** | -1.287*** |
|  | (.194) | (.125) | (.16) | (.15) | (.121) | (.147) |
| U0 x (e = 4) | -.164 | -.446*** | -.839*** | -.308*** | -.628*** | -.947*** |
|  | (.127) | (.074) | (.125) | (.098) | (.083) | (.121) |
| U0 x (e = 9) | .237** | .161** | .185 | .126 | .163** | .006 |
|  | (.094) | (.066) | (.186) | (.096) | (.077) | (.176) |
| Constant | 179.963*** | 187.483*** | 285.032*** | 129.383*** | 142.848*** | 280.244*** |
|  | (12.896) | (6.885) | (9.829) | (9.824) | (10.225) | (12.064) |
| Observations | 7850 | 9459 | 6558 | 8674 | 9596 | 6994 |
| R-squared | .483 | .619 | .259 | .539 | .674 | .279 |
| \| **Model 2. Interactions of initial unemployment with experience and trends** \| \| --- \| | | | | | | |
| U0 x (e = 0) | .366* | -.24** | -.571*** | .195 | -.113 | -.595*** |
|  | (.188) | (.119) | (.146) | (.14) | (.125) | (.139) |
| U0 x (e = 4) | -.267** | -.402*** | -.384*** | -.421*** | -.595*** | -.415*** |
|  | (.123) | (.079) | (.115) | (.1) | (.094) | (.125) |
| U0 x (e = 9) | .055 | .097 | .16 | -.097 | .099 | .04 |
|  | (.096) | (.074) | (.163) | (.098) | (.087) | (.148) |
| Year trend x (e = 0) | -2.821*** | -1.94*** | -3.48*** | -2.858*** | -2.43*** | -3.886*** |
|  | (.375) | (.216) | (.356) | (.343) | (.211) | (.341) |
| Year trend x (e = 4) | -.936*** | -.503*** | -2.309*** | -1.211*** | -.296* | -2.353*** |
|  | (.287) | (.152) | (.265) | (.211) | (.175) | (.26) |
| Year trend x (e = 9) | -1.001*** | -.463*** | -1.759*** | -1.294*** | -.453** | -2.5*** |
|  | (.19) | (.165) | (.304) | (.214) | (.188) | (.352) |
| Constant | 230.01*** | 223.264*** | 356.333*** | 178.493*** | 185.506*** | 355.938*** |
|  | (13.98) | (8.063) | (11.826) | (12.804) | (10.534) | (12.639) |
| Observations | 7850 | 9459 | 6558 | 8674 | 9596 | 6994 |
| R-squared | .503 | .636 | .349 | .571 | .695 | .375 |
| **Model 4: Mode 3 + fixed effect for year of graduation + National unemployment rate** | | | | | | |
| U0 x (e = 0) | .746*** | .064 | -.284 | .615*** | .419*** | -.32* |
|  | (.199) | (.137) | (.177) | (.15) | (.127) | (.171) |
| U0 x (e = 4) | -.113 | -.264*** | -.218 | -.194* | -.308*** | -.279* |
|  | (.131) | (.093) | (.161) | (.112) | (.087) | (.149) |
| U0 x (e = 9) | 0 | .123 | .08 | -.035 | .241** | .089 |
|  | (.118) | (.091) | (.209) | (.117) | (.107) | (.177) |
| Year trend x (e = 0) | -3.248*** | -1.879*** | -2.923*** | -3.646*** | -1.497** | -2.229*** |
|  | (.586) | (.5) | (.414) | (.548) | (.66) | (.557) |
| Year trend x (e = 4) | -1.043* | -.533 | -1.857*** | -1.584*** | .506 | -.656 |
|  | (.548) | (.507) | (.517) | (.503) | (.662) | (.548) |
| Year trend x (e = 9) | -.503 | -.363 | -2.042*** | -1.192** | .394 | -1.083* |
|  | (.568) | (.539) | (.61) | (.526) | (.704) | (.582) |
| National UR | -1.576*** | -.91*** | -.692*** | -1.342*** | -1.06*** | -.604*** |
|  | (.156) | (.133) | (.178) | (.144) | (.12) | (.177) |
| Constant | 266.759*** | 247.898*** | 331.172*** | 222.285*** | 208.128*** | 324.811*** |
|  | (15.665) | (9.179) | (12.229) | (12.798) | (10.623) | (15.337) |
| Observations | 7850 | 9459 | 6558 | 8674 | 9596 | 6994 |
| R-squared | .53 | .655 | .373 | .598 | .714 | .396 |

Note. The sample period is 1987-2019. Models 1 and 2 include fixed effects for the province of birth and years elapsed since graduation. Model 3 adds graduation cohort. Standard errors are in parentheses. *** *p*<.01, ** *p*<.05, * *p*<.10.

**Table A5. Model 3 estimates of scarring and trend effects. Region of birth**

|  | **Log real monthly earnings** | | | **Log real daily wages** | | | |
| --- | --- | --- | --- | --- | --- | --- | --- |
|  | Junior college | College | Graduate | Junior college | College | Graduate | |
| U0 x (e = 0) | .01** | .001 | -.004 | .008** | -.002 | 0 | |
|  | (.004) | (.003) | (.005) | (.003) | (.002) | (.003) | |
| U0 x (e = 1) | .007 | -.007*** | -.007 | .004 | -.005*** | -.002 | |
|  | (.004) | (.002) | (.005) | (.003) | (.002) | (.003) | |
| U0 x (e = 2) | 0 | -.011*** | -.012*** | .001 | -.007*** | -.005* | |
|  | (.004) | (.002) | (.004) | (.003) | (.002) | (.003) | |
| U0 x (e = 3) | -.001 | -.011*** | -.008* | -.001 | -.007*** | -.003 | |
|  | (.004) | (.002) | (.004) | (.003) | (.002) | (.003) | |
| U0 x (e = 4) | -.001 | -.009*** | -.007 | -.002 | -.006*** | -.001 | |
|  | (.004) | (.002) | (.005) | (.003) | (.001) | (.003) | |
| U0 x (e = 5) | .001 | -.007*** | -.002 | -.003 | -.007*** | .001 | |
|  | (.003) | (.002) | (.004) | (.003) | (.001) | (.002) | |
| U0 x (e = 6) | -.001 | -.007*** | .002 | -.003 | -.006*** | .001 | |
|  | (.003) | (.002) | (.004) | (.003) | (.002) | (.002) | |
| U0 x (e = 7) | .001 | -.008*** | .004 | -.003 | -.008*** | .002 | |
|  | (.004) | (.002) | (.005) | (.003) | (.002) | (.003) | |
| U0 x (e = 8) | .001 | -.009*** | .001 | -.003 | -.007*** | -.001 | |
|  | (.004) | (.003) | (.005) | (.003) | (.002) | (.003) | |
| U0 x (e = 9) | .002 | -.006** | -.004 | -.002 | -.007*** | -.003 | |
|  | (.004) | (.003) | (.005) | (.003) | (.002) | (.003) | |
| Year trend x (e = 0) | -.028*** | -.025*** | -.056*** | .001 | -.008*** | -.03*** | |
|  | (.002) | (.001) | (.006) | (.002) | (.001) | (.004) | |
| Year trend x (e = 1) | -.027*** | -.024*** | -.046*** | -.001 | -.006*** | -.028*** | |
|  | (.002) | (.001) | (.006) | (.002) | (.001) | (.003) | |
| Year trend x (e = 2) | -.023*** | -.022*** | -.04*** | -.003** | -.006*** | -.023*** | |
|  | (.002) | (.001) | (.006) | (.001) | (.001) | (.003) | |
| Year trend x (e = 3) | -.022*** | -.018*** | -.036*** | -.005*** | -.006*** | -.02*** | |
|  | (.002) | (.001) | (.006) | (.001) | (.001) | (.003) | |
| Year trend x (e = 4) | -.019*** | -.015*** | -.032*** | -.004*** | -.007*** | -.018*** | |
|  | (.002) | (.001) | (.006) | (.001) | (.001) | (.003) | |
| Year trend x (e = 5) | -.018*** | -.014*** | -.03*** | -.004*** | -.006*** | -.017*** | |
|  | (.002) | (.001) | (.006) | (.001) | (.001) | (.003) | |
| Year trend x (e = 6) | -.016*** | -.014*** | -.029*** | -.004*** | -.006*** | -.017*** | |
|  | (.002) | (.001) | (.006) | (.001) | (.001) | (.003) | |
| Year trend x (e = 7) | -.015*** | -.013*** | -.028*** | -.004** | -.005*** | -.015*** | |
|  | (.002) | (.001) | (.006) | (.002) | (.001) | (.004) | |
| Year trend x (e = 8) | -.016*** | -.014*** | -.029*** | -.005*** | -.006*** | -.015*** | |
|  | (.002) | (.001) | (.006) | (.001) | (.001) | (.004) | |
| Year trend x (e = 9) | -.016*** | -.014*** | -.03*** | -.006*** | -.007*** | -.016*** | |
|  | (.002) | (.002) | (.006) | (.002) | (.001) | (.004) | |
| National UR | -.008*** | -.007*** | -.003** | -.005*** | -.005*** | -.002*** | |
|  | (.001) | (.001) | (.001) | (.001) | (.001) | (.001) | |
| Constant | 6.291*** | 6.483*** | 7.825*** | 2.976*** | 3.269*** | 4.113*** | |
|  | (.078) | (.06) | (.222) | (.055) | (.039) | (.125) | |
| Observations | 3861 | 4125 | 3579 | 3861 | 4125 | 3578 | |
| R-squared | .788 | .887 | .792 | .696 | .85 | .777 | |
|  | | | | | | |  |

**Table A5. Model 3 estimates of scarring and trend effects. Region of birth (continued)**

|  | **Annual days worked by match** | | | **Annual days worked by worker** | | | |
| --- | --- | --- | --- | --- | --- | --- | --- |
|  | Junior college | College | Graduate | Junior college | College | Graduate | |
| U0 x (e = 0) | 2.217*** | 1.295*** | -.341 | 2.464*** | 1.294*** | -.401 | |
|  | (.664) | (.336) | (.558) | (.683) | (.379) | (.528) | |
| U0 x (e = 1) | 1.192* | .204 | -1.083** | 1.253* | .055 | -.997** | |
|  | (.62) | (.31) | (.536) | (.643) | (.345) | (.5) | |
| U0 x (e = 2) | .373 | -.663** | -1.717*** | -.043 | -.675** | -1.152** | |
|  | (.584) | (.305) | (.51) | (.59) | (.32) | (.49) | |
| U0 x (e = 3) | -.189 | -1.017*** | -1.46*** | -.464 | -.759** | -1.316*** | |
|  | (.572) | (.322) | (.517) | (.568) | (.324) | (.448) | |
| U0 x (e = 4) | -.471 | -1.269*** | -1.104** | -.56 | -.812** | -.648 | |
|  | (.549) | (.326) | (.54) | (.536) | (.319) | (.48) | |
| U0 x (e = 5) | -.367 | -1.057*** | -.865 | -.61 | -.786** | -.593 | |
|  | (.511) | (.326) | (.535) | (.506) | (.305) | (.469) | |
| U0 x (e = 6) | -.497 | -1.034*** | -.429 | -.561 | -.501* | -.187 | |
|  | (.532) | (.345) | (.571) | (.522) | (.3) | (.482) | |
| U0 x (e = 7) | -.182 | -.867** | -.28 | -.567 | -.396 | -.238 | |
|  | (.561) | (.374) | (.629) | (.544) | (.328) | (.475) | |
| U0 x (e = 8) | .063 | -.351 | -.506 | -.121 | .233 | -.238 | |
|  | (.607) | (.438) | (.728) | (.567) | (.376) | (.56) | |
| U0 x (e = 9) | .108 | .32 | -.762 | -.102 | .615 | -.124 | |
|  | (.62) | (.437) | (.659) | (.638) | (.39) | (.589) | |
| Year trend x (e = 0) | -5.074*** | -3.985*** | -5.863*** | -4.634*** | -3.347*** | -4.903*** | |
|  | (.348) | (.186) | (.689) | (.345) | (.192) | (.522) | |
| Year trend x (e = 1) | -4.563*** | -3.429*** | -5.413*** | -3.681*** | -2.461*** | -3.815*** | |
|  | (.308) | (.163) | (.612) | (.288) | (.165) | (.447) | |
| Year trend x (e = 2) | -3.934*** | -3.203*** | -4.595*** | -2.814*** | -1.897*** | -3.116*** | |
|  | (.274) | (.159) | (.669) | (.281) | (.16) | (.499) | |
| Year trend x (e = 3) | -3.748*** | -3.051*** | -4.58*** | -2.364*** | -1.629*** | -3.001*** | |
|  | (.264) | (.176) | (.669) | (.27) | (.165) | (.462) | |
| Year trend x (e = 4) | -3.203*** | -2.748*** | -5.082*** | -1.941*** | -1.465*** | -3.437*** | |
|  | (.253) | (.172) | (.676) | (.252) | (.161) | (.465) | |
| Year trend x (e = 5) | -3.448*** | -2.752*** | -5.157*** | -2.079*** | -1.305*** | -3.148*** | |
|  | (.244) | (.182) | (.742) | (.251) | (.168) | (.51) | |
| Year trend x (e = 6) | -3.254*** | -2.657*** | -5.147*** | -1.958*** | -1.359*** | -3.508*** | |
|  | (.262) | (.198) | (.709) | (.24) | (.17) | (.492) | |
| Year trend x (e = 7) | -3.088*** | -2.851*** | -4.84*** | -1.744*** | -1.589*** | -3.23*** | |
|  | (.314) | (.211) | (.736) | (.283) | (.184) | (.509) | |
| Year trend x (e = 8) | -3.027*** | -2.86*** | -5.269*** | -1.777*** | -1.467*** | -3.767*** | |
|  | (.303) | (.25) | (.721) | (.272) | (.208) | (.521) | |
| Year trend x (e = 9) | -3.15*** | -2.835*** | -5.575*** | -1.685*** | -1.541*** | -3.898*** | |
|  | (.36) | (.295) | (.758) | (.361) | (.264) | (.562) | |
| National UR | .027 | .368*** | .742*** | -1.611*** | -1.15*** | -.613*** | |
|  | (.182) | (.131) | (.177) | (.165) | (.131) | (.152) | |
| Constant | 146.43*** | 154.934*** | 289.05*** | 210.064*** | 216.551*** | 357.745*** | |
|  | (11.94) | (8.076) | (26.216) | (12.095) | (7.886) | (19.241) | |
| Observations | 3861 | 4125 | 3579 | 3861 | 4125 | 3579 | |
| R-squared | .785 | .865 | .704 | .824 | .89 | .67 | |
| \| Note. The sample period is 1987-2019 for college and graduate degrees. For junior college workers it is 1987-2013. The model includes fixed effects for the region of birth, years elapsed since graduation, and graduation cohort. Standard errors are in parentheses. *** *p*<.01, ** *p*<.05, * *p*<.10. \| \| --- \| | | | | | | |  |
